# Dufulin Activates HrBP1 to Produce Antiviral Responses in Tobacco

**DOI:** 10.1371/journal.pone.0037944

**Published:** 2012-05-25

**Authors:** Zhuo Chen, Mengjiao Zeng, Baoan Song, Chengrui Hou, Deyu Hu, Xiangyang Li, Zhenchao Wang, Huitao Fan, Liang Bi, Jiaju Liu, Dandan Yu, Linhong Jin, Song Yang

**Affiliations:** 1 State Key Laboratory Breeding Base of Green Pesticide and Agricultural Bioengineering, Key Laboratory of Green Pesticide and Agricultural Bioengineering, Ministry of Education, Guizhou University, Guiyang, China; 2 School of Chemistry and Chemical Engineering, South China University of Technology, Guangzhou, China; 3 Shanghai Applied Protein Technology Co. Ltd., Shanghai, China; Drexel University College of Medicine, United States of America

## Abstract

**Background:**

Dufulin is a new antiviral agent that is highly effective against plant viruses and acts by activating systemic acquired resistance (SAR) in plants. In recent years, it has been used widely to prevent and control tobacco and rice viral diseases in China. However, its targets and mechanism of action are still poorly understood.

**Methodology/Principal Findings:**

Here, differential in-gel electrophoresis (DIGE) and classical two-dimensional electrophoresis (2-DE) techniques were combined with mass spectrometry (MS) to identify the target of Dufulin. More than 40 proteins were found to be differentially expressed (≥1.5 fold or ≤1.5 fold) upon Dufulin treatment in *Nicotiana tabacum* K_326_. Based on annotations in the Gene Ontology (GO) and the Kyoto Encyclopedia of Genes and Genomes (KEGG) databases, these proteins were found to be related to disease resistance. Directed acyclic graph (DAG) analysis of the various pathways demonstrated harpin binding protein-1 (HrBP1) as the target of action of Dufulin. Additionally, western blotting, semi-quantitative reverse transcription polymerase chain reaction (RT-PCR), and real time PCR analyses were also conducted to identify the specific mechanism of action of Dufulin. Our results show that activation of HrBP1 triggers the salicylic acid (SA) signaling pathway and thereby produces antiviral responses in the plant host. A protective assay based on lesion counting further confirmed the antiviral activity of Dufulin.

**Conclusion:**

This study identified HrBP1 as a target protein of Dufulin and that Dufulin can activate the SA signaling pathway to induce host plants to generate antiviral responses.

## Introduction

Harpin binding protein-1 (HrBP1), which is found in plant cell walls, induces plants to generate systemic acquired resistance (SAR) and has important biological significance for pest control [1]. HrBP1 was first discovered in plant cell walls while studying the biological activity of harpin, a protein secreted by Gram-negative bacteria and other microorganisms and capable of inducing hypersensitive response [1]. HrBP1 was subsequently detected in *Arabidopsis thaliana,* soybean, barley, tomatoes, rice, potatoes, wheat, corn, and other plants. It is encoded by 284 amino acids and has a molecular weight (MW) of approximately 30 kDa with conserved primary structures in many plants [1]. In its activated state, HrBP1 can selectively up-regulate several signaling pathways and generate anti-bacterial, anti-viral, anti-fungal and anti-insect responses in the plant host. Independent signaling pathways induced by HrBP1 include the *enhanced disease susceptibility-5* (*EDS5*)*-*salicylic acid (SA)/*nonexpressor of pathogenesis-related genes 1*(*NPR1*)/unknown effective protein pathway, the EDS/SA/NPR1/AOX/PR pathway, and the ethylene (ET)/jasmonic acid (JA)/PDF1.2/Thi2.1 pathway. Some studies have shown that changes in the activation state of HrBP1 are caused by changes in its spatial structure, which can vary depending on the ligand [2]–[5]. HrBP1 has also been shown to up-regulate *Harpin-induced gene* (*H1N1)*, a newly discovered pathogenesis-related gene, which induces mitogen-activated protein kinase (MAPK) activity to generate disease resistance [6]. Moreover, production of hypersensitivity reaction (HR) and SAR via the harpin–HrBP1 interaction has been found to be closely associated with the *nonrace-specific disease resistance* (*NDR1*) and *enhanced disease susceptibility-1* (*EDS1)* genes [7], and to be inhibited by exogenous auxin [8].

**Figure 1:**
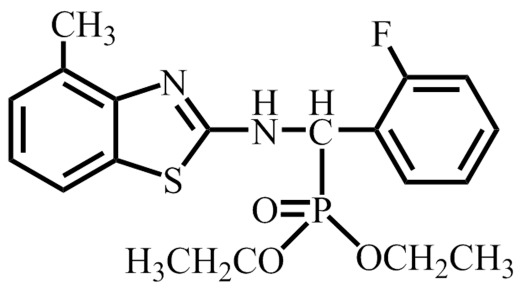
Molecular structure of Dufulin.

Dufulin, an amino phosphonate compound, is a plant antiviral agent with a novel molecular structure and good antiviral activity against tobacco mosaic virus (TMV), cucumber mosaic virus, and Potato Virus Y, among many other plant viruses (Figure 1). Preliminary studies have shown that it exerts its antiviral effects by activating SAR, but the specific mechanisms of its action and its molecular targets remain unclear [9]. In the present study, differential in-gel electrophoresis (DIGE)/two-dimensional electrophoresis (2-DE) combined with mass spectrometry (MS), bioinformatics, western blotting and reverse transcription polymerase chain reaction (RT-PCR) was used to identify the target of action of Dufulin. Its antiviral activity was found to involve SAR, and HrBP1 was identified as its target protein. Based on the current understanding of HrBP1 and the downstream regulatory pathways associated with its antiviral functions, the expression levels of some key genes such as *EDS* and *pathogenesis-related protein* (*PR*) were assessed using RT-PCR in plants treated with Dufulin. Our results validate HrBP1 as the target of action of Dufulin.

## Results

### 2D-DIGE Analysis

**Figure 2:**
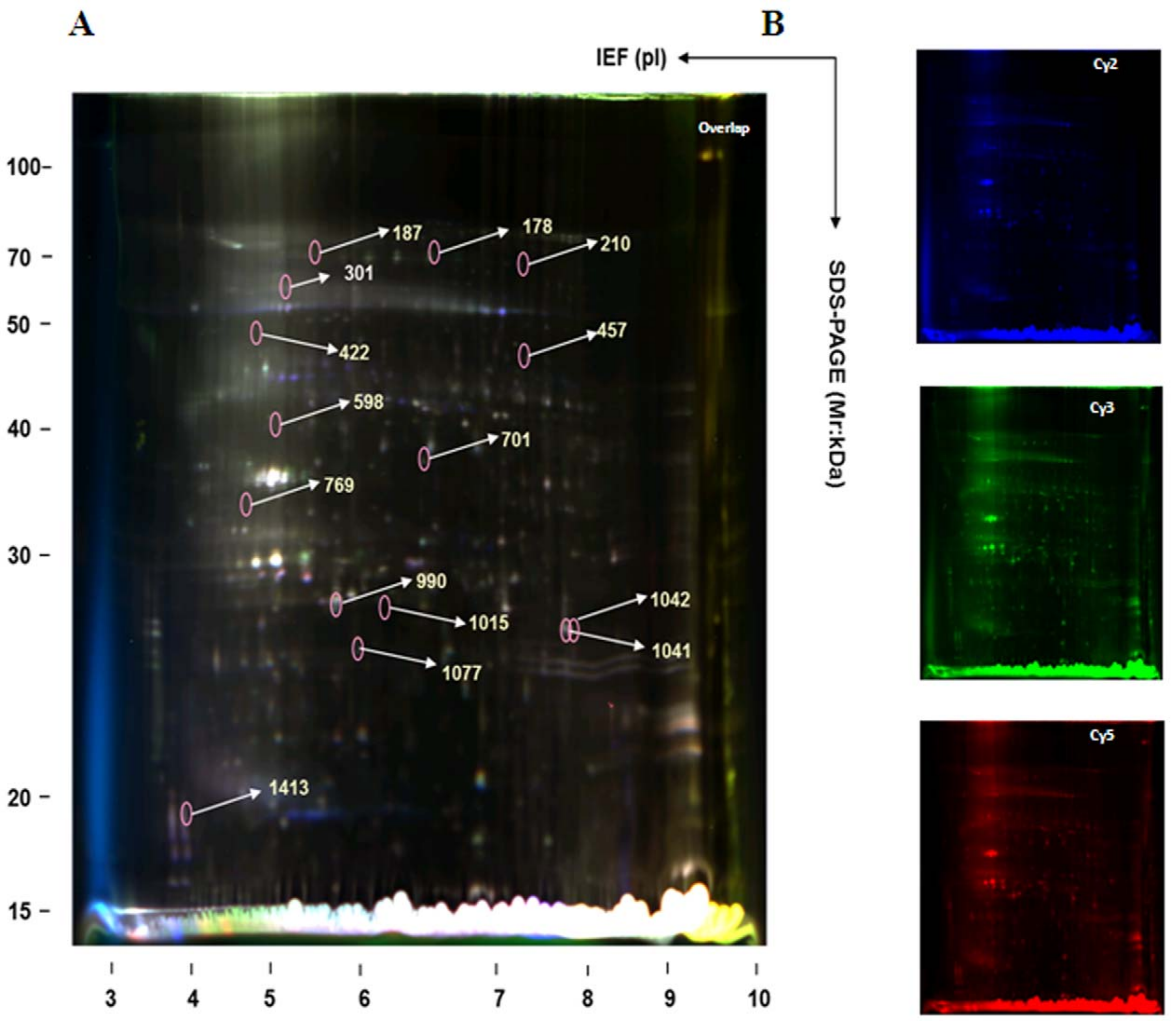
2D-DIGE image analysis of total proteins from the tobacco leaves of Groups B–E. (**A**) Composite image of Cy2, Cy3 and Cy5 images. (**B**), (**C**), and (**D**) represent the Cy2, Cy3 and Cy5 fluorescent dye images, respectively.

**Figure 3:**
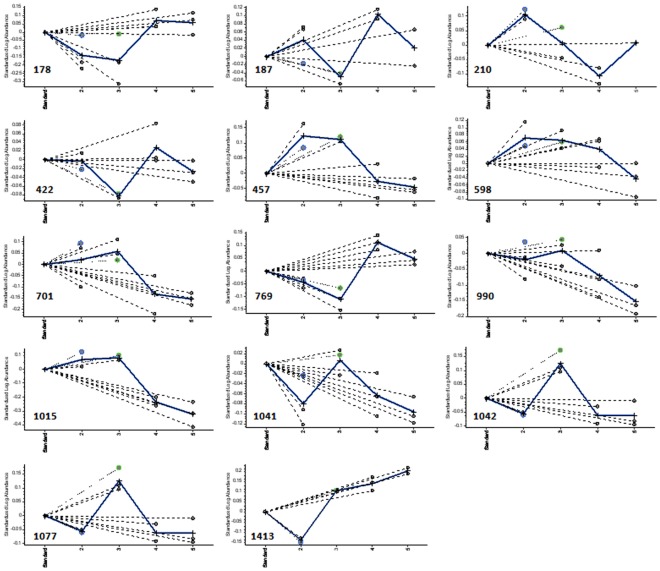
Expression levels and differential expression of some proteins. Each part of the figure shows the standard log abundance of spots on the *y*-axis for each protein spot of Groups B, C, D, and E (pool groups 2, 3, 4, and 5, respectively) on the *x*-axis. The dotted line represents the mean abundance for three repeated measurements in Groups B–D, whereas the continuous line represents the mean abundance for three repeated treatments in any single treatment group.

Samples from all treatment groups were labeled with fluorescent dyes (Cy2, Cy3, or Cy5) [10], [11] and run on the following conditions to eliminate their inherent biological and systemic variations: (1) the same sample was divided into two parts, labeled with Cy3 and Cy5, and run on two gels; (2) different treatment groups from the same plant were not run on the same gel; (3) two samples within the same gel were placed as far apart as possible; and (4) internal standards were mixed with equal amounts of all samples, labeled with Cy2, and run on each gel [10], [11] (Figure 2). In order to eliminate differences between gels and improve the statistical reliability of our data, the value of each protein spot was calculated as the ratio of the measured values of the protein spot to the total value from all protein spots in the internal standard group. In addition, the same protein labeled with all three fluorescent dyes showed identical protein spots at the same position in the gel, thereby enhancing the matching rate of the protein spots. DeCyder software was used for image analysis to avoid errors caused by the user’s subjective influence in the selection of points. Protein points without appropriate matches were considered to arise from impurities or random errors in the experiment. A total of 14 protein spots showed statistically significant changes in expression across the four treatment groups (B, C, D, and E; one-way ANOVA, *P*<0.05). These spots were mainly within the pH range of 5–8 (Figure 3).

The 14 protein spots were identified as belonging to 13 protein products using matrix-assisted laser desorption/ionization tandem time-of-flight (MALDI-TOF/TOF) MS and linear trap quadrupole (LTQ) MS (Table 1, Table 2 and Table S1). The software could not search for corresponding information related to tobacco likely due to lack of information on plant proteins in the database. Of the 14 protein spots, 1 protein (spot 457) was annotated as “putative *β*-galactosidase” from a different species (*Solanum lycopersicum*); another protein (spot 310) was designated as the CF1-α subunit of ATP synthase from yet another species (*Nicotiana sylvestris*). The trends in changes in protein expression across the four treatment groups are illustrated in Figure 3. Spots 187, 769, 990, and 1041 were ascribed to genes associated with disease resistance and were upregulated in groups C and D. Four other proteins (spots 210, 422, 701, and 1413) identified were found to control the biological functions of carbon metabolism, photosynthesis, as well as photorespiration and cell division. Spots 457 (putative *β*-galactosidase) and 598 (carboxylase/oxygenase) showed trends towards downregulation.

**Table 1:** Differentially expressed proteins identified by DIGE and MS.

DIGE spot no.	Protein description	Species	GI no.	Function	Subcellular location	Theoretical MW (Da)/pI	ExperimentalMW (kDa)/pI	Peptide Count	Protein Score	Protein ScoreC.I %
301	ATP synthase CF1-α subunit	*Nicotiana sylvestris*	gi|78102516	energy metabolism	mitochondrial membranes	55388/5.14	62.4/5.15	19	686	100
178	Chain A of ribulose-1,5-bisphosphate carboxylase oxygenase	*N. tabacum*	gi|515239	photosynthesis and photorespiration	chloroplast	49526.1/6.19	70.1/6.57	14	250	100
457	putative β-galactosidase	*Solanum lycopersicum*	gi|7939623	carbon metabolism	lysosome	93183.9/6.81	46.9/7.28	11	339	100
598	carboxylase/oxygenase	*N. tabacum*	gi|223593	carbon metabolism	cytoplasm	14490.2/4.99	40.5/5.05	5	65	78.524
769	HrBP1	*N. tabacum*	gi|38679323	disease resistance	cell membrane	29980/8.8	34.4/4.66	8	242	100
990	germin-like protein	*N. tabacum*	gi|222051768	disease resistance	cytoplasm	21408.2/5.84	28.2/5.77	4	257	100
1015	oxygen-evolving complex33-kDa photosystem II	*N. tabacum*	gi|30013657	photosynthesis and photorespiration	chloroplast	35176.9/5.63	27.9/6.17	11	284	100
1041	24 K germin-like protein	*N. tabacum*	gi|31711507	disease resistance	cytoplasm	21954.4/7.82	27.1/7.78	4	350	100
187	truncated N-protein	*N. tabacum*	gi|45544515	disease resistance	cytoplasm	74429	70.15/5.53	30	4.9e+02	4
210	putative cell cycle protein	*N. tabacum*	gi|82775180	cell division	nucleus	20523	68.55/7.28	22	2.8e+03	2

**Table 2:** Differentially expressed proteins identified by DIGE and LTQ spectrometry.

DIGE spot no.	Protein Description	Species	GI no.	Function	Subcellular location	Peptide count	Unique peptide count	CoverPercent	MW	PI	XC	DeltaCn
						Peptide Sequence	MH+	Diff(MH+)	Charge	Rank		
422	ribulose-bisphosphate carboxylase activase	*N. tabacum*	gi|19992	photosynthesis and photorespiration	Chloroplast	4	2	8.19%	25929.41	5.01		
						R.TDNVPEEAVVK.I	1201.309	0.39451	2	1	2.4651	0.6007
						R.TDNVPEEAVVK.I	1201.309	0.48751	2	1	2.4786	0.5422
						R.VYDDEVRK.W	1024.109	–0.41359	2	1	2.6064	0.5159
						R.VYDDEVRK.W	1024.109	0.18941	2	1	2.2002	0.4997
701	Phosphomannomutase	*N. tabacum*	gi|90762161	carbon metabolism	Cytoplasm	1	1	4.37%	28575.43	6.41		
						R.SGM*LNVSPIGR.D	1147.331	–0.93531	2	1	2.4934	0.5942
1042	24 K germin-likeprotein	*N. tabacum*	gi|31711507	disease resistance	Cytoplasm	1	1	2.86%	21968.49	5.83		
						K.LNPLIK.A	697.889	0.18904	1	1	2.1532	0.3057
1413	ribulose bisphosphate carboxylase small-subunit precursor	*N. tabacum*	gi|30013663	photosynthesis and photorespiration	Chloroplast	2	1	5.00%	20281.19	7.57		
						R.GFVYRENNK.S	1127.235	–0.58645	2	1	2.5666	0.539
						R.GFVYRENNK.S	1127.235	0.29255	2	1	2.6403	0.4976

### Classic 2-DE Analysis

**Figure 4:**
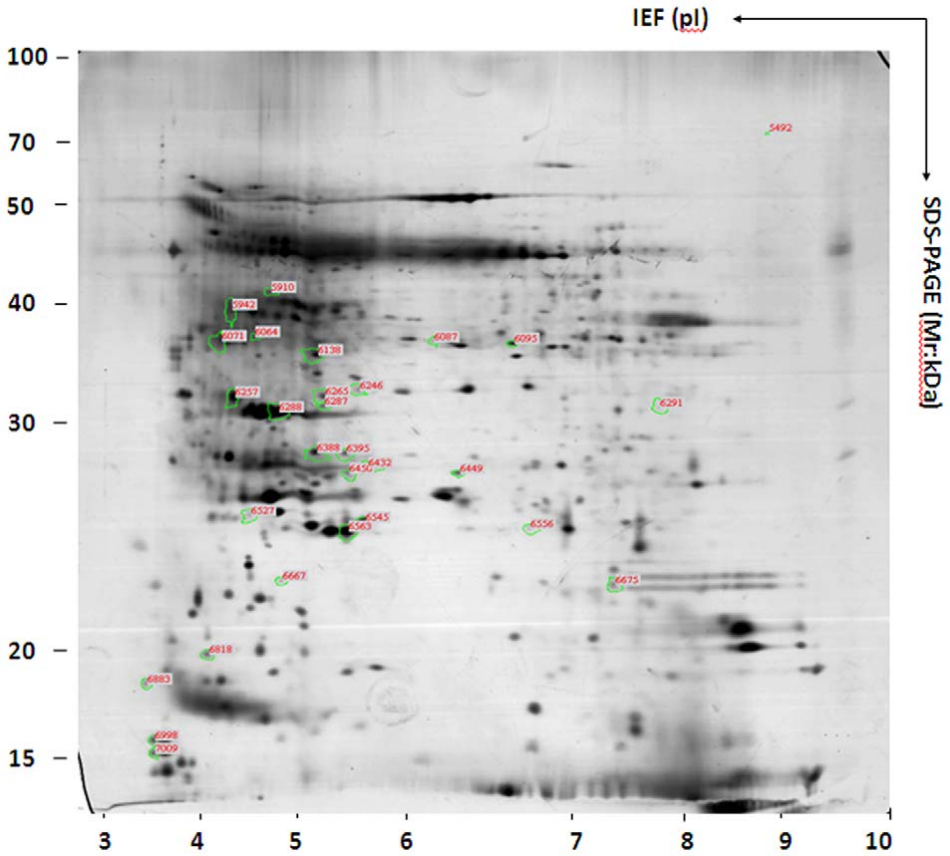
2-DE image analysis of total proteins from the tobacco leaves of Groups E. The spots were visualized by silver staining and detected using the DeCyder software. Quantitative image analysis revealed the protein spots with significantly different intensity (*p<0.05*) (by more than 1.5-fold) when compared with the B group.

In order to make up for the shortcoming of the DIGE assay, which involves lower loading quantities than other methods, 2-DE analysis combined with silver staining and coomassie brilliant blue staining was performed in three replicates on group B (tobacco infected by TMV for 1 day) and E (tobacco treated with Dufulin for 5 day in group B). The goal of these experiments was to identify some low-abundance proteins by loading 800 µg of sample in each well [12], [13], and to investigate temporal changes in protein profiles that occur during the infection of tobacco leaves by TMV and after their 5-day treatment with Dufulin (group E). Each sample was run in gels in triplicates, and they showed a high degree of reproducibility (Figure 4). Our quantitative image analysis showed 21 protein spots whose intensities changed (*P<*0.05) by at least 1.2-fold (Table S2 and Figure S1). The molecular weights of the differentially expressed proteins were mainly in areas corresponding to the MW markers 20–40 kDa. Intensities of the protein spots in group E were as high as 79.3% relative to the group B, and their pH ranged from 4–7.

In total, 21 spots representing 20 proteins were successfully identified using MALDI-TOF/TOF MS (Table 3 and Table S3). Four proteins were annotated either as unknown and hypothetical proteins, or as proteins without any specific function assigned in the database; other proteins were identified to be associated with photosynthesis, photorespiration, disease resistance, stress response, as well as the metabolism of redox components, lipids, and energy. For example, the spots 5942, 6138, 6087, 6288 6291, 6527, 6556, and 6675 were identified as proteins associated with photorespiration and photosynthesis, while the spots 6071, 6257, and 6388 were identified as being associated with disease resistance. Interestingly, two spots (6388 and 6395) were identified as HrBP1, which was also identified by DIGE/MS to have a trend of up-regulation (Figure 3). A comparison of our results of the 2-DE and DIGE assays showed that four proteins [GenInfo identifier (GI) nos. 100380, 515239, 30013663, and 38679323] were identified in both assays, indicating that our 2-DE assays complemented the DIGE assay well. However, some proteins related to disease resistance (spots 6071 and 6257) were identified by 2-DE/MS, but not by DIGE/MS, indicating that the results of the two assays don’t correspond fully with each other.

**Table 3:** Differentially expressed proteins identified by 2-DE and MS.

Spot no.	Protein description	Species	GI no.	Function	Subcellular location	TheoreticalMw (Da)/pI	Experimental Mw (kDa)/pI	Peptide count	Protein Score	Protein ScoreC.I %	Best Ion Score	ratio	Regulation tendency
5942	ribulose bisphosphate carboxylase/oxygenase-2 (RuBisCO activase)	*N. tabacum*	gi|12643758	photosynthesis and photorespiration	chloroplast	48312.5/8.14	39.14/4.40	9	217	100	76	1.28723	down
6071	lignin-forming anionic peroxidase	*N. tabacum*	gi|129837	disease resistance	cytoplasm	34652.1/4.69	36.24/4.25	8	260	100	142	2.31966	down
6138	Photosystem-II stability/assembly factor HCF 136	Ricinus communis	gi|255559812	photosynthesis and photorespiration	chloroplast	43414.9/7.11	36.01/5.40	9	209	100	99	1.28514	down
6087	ribulose-bisphosphate carboxylase activase	*N. tabacum*	gi|100380	photosynthesis and photorespiration	chloroplast	25913/5.01	36.67/6.25	8	302	100	117	1.31604	up
6095	NAD-dependent epimerase/dehydratase	Ricinus communis	gi|255542956	redox metabolism	cytoplasm	42547/8.52	66.35/6.75	8	364	100	110	1.6325	up
6257	plastid-lipid-associated protein	*N. tabacum*	gi|2632088	Lipid metabolism stress response	chloroplast	29385.4/4.83	32.00/4.55	8	377	100	89	1.98999	up
6288	oxygen-evolving complex 33-kDa photosystem-II protein	*N. tabacum*	gi|30013657	photosynthesis and photorespiration	chloroplast	35176.9/5.63	30.67/4.90	15	653	100	170	1.29081	up
6265	predicted protein	Micromonas pusilla	gi|226461019	No annotation	No annotation	272410.5/5.67	32.67/5.44	32	75	97.645		1.49341	down
6287	protein-binding protein	Ricinus communis	gi|255564826	No annotation	No annotation	37785.1/7.56	31.33/5.48	8	136	100	60	1.31604	down
6246	predicted protein	Physcomitrella patens subsp. Patens	gi|168050023	No annotation/	No annotation	70926.8/9.06	33.34/5.78	19	78	98.972		1.49111	down
6291	chain A of ribulose-1,5-Bisphosphate carboxylase oxygenase	*N. tabacum*	gi|515239	photosynthesis and photorespiration	chloroplast	49526.1/6.19	31.15/7.84	11	85	99.78	27	1.24821	up
6388	HrBP1	*N. tabacum*	gi|38679323	disease resistance	cell membrane	29980/8.8	34.4/4.66	8	197	100	73	1.36946	
6527	oxygen-evolvingenhancer protein 2-1	*N. tabacum*	gi|52000814	photosynthesis and photorespiration	chloroplast	28634.4/6.84	26.16/4.75	7	315	100	109	1.44689	up
6563	superoxide dismutase	Nicotiana plumbaginifolia	gi|134642	disease resistance	chloroplast	23027.5/5.53	25.40/5.66	9	323	100	78	1.35317	down
6545	predicted protein	Populustrichocarpa	gi|224104053	No annotation	No annotation	70270.2/7.85	25.81/5.74	14	64	72.963	6	1.27646	down
6449	cytosolic isoform-liketriose phosphate isomerase	Solanumtuberosum	gi|77745458	energy metabolism	cytoplasm	26995/5.73	27.75/6.40	9	376	100	102	1.77561	up
6556	light-harvestingcomplex I protein Lhca1	Populustrichocarpa	gi|224109746	photosynthesis and photorespiration	chloroplast	26319.5/5.83	25.46/6.90	2	58	0	50	1.28703	up
6675	PSI-D1 precursor	*Nicotiana sylvestris*	gi|407769	photosynthesis and photorespiration	thylakoid membrane	23441.2/9.84	23.05/7.46	9	106	99.998	45	1.50195	down
6395	HrBP1	*N. tabacum*	gi|38679323	disease resistance	cell membrane	29980/8.8	28.55/5.66	9	201	100	66	2.05393	down
6818	chain ‘s’ of ribulose-1,5-bisphosphate carboxylase oxygenase	*N. tabacum*	gi|230922	photosynthesis and photorespiration	chloroplast	14550.2/5.19	19.95/4.15	7	213	100	68	1.6636	up
6883	putative ribulose bisphosphate carboxylase small subunit precursor protein	*N. tabacum*	gi|30013663	photosynthesis and photorespiration	chloroplast	20268.1/7.57	18.75/3.55	7	162	100	27	1.38414	up

### Upregulation of the SA-mediated Signaling Pathway Identified Using Bioinformatic Analysis of Proteomic Data

**Figure 5:**
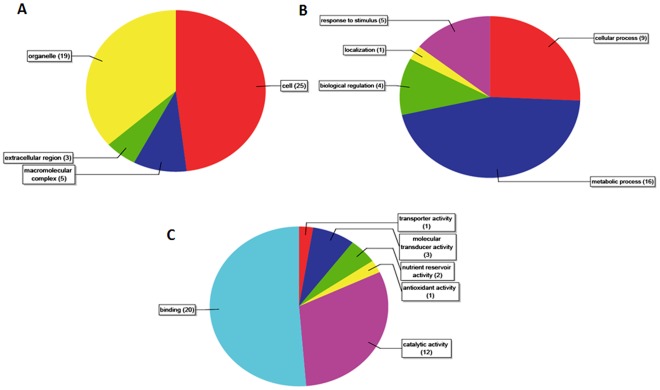
Pie graphs illustrating the distribution of target sequences based on their annotation in KEGG. (**A**) Based on their annotated molecular function (MF), the genes were classified as “transporter activity”, “molecular transducer activity”, “nutrient reservoir activity”, or “catalytic activity”. (**B**) Based on their annotated involvement in various biological processes (BP), genes were classified as “response to stimulus”, “localization”, “biological regulation”, “cellular process”, and “metabolic process”. (**C**) Based on their annotated localization in various cellular components (CC), genes were classified as “cell”, “organelle”, and “macromolecular complex”.

**Figure 6:**
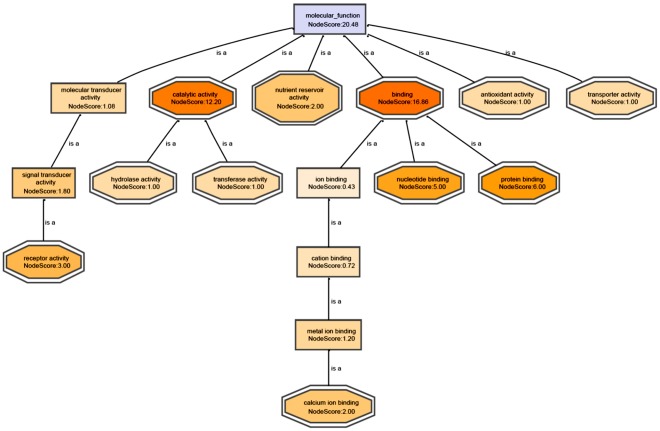
Pathway analysis of the target of Dufulin in mediating anti-viral responses using MF-DAG. Nodes are colored according to their score values. The DAGs of BP and CC are shown in Figures S7 and S8.

The wealth of discrete proteomic data available today has made their bioinformatic analysis both necessary and feasible. To this end, proteins that were found to have differential expression in our proteomics assay were annotated based on the Genbank database. Excluding data for 13 redundant sequences and 2 sequences that could not be retrieved (accession codes 76869447 and 51964984), information related to 33 sequences was downloaded from GenBank and the sequences were analyzed using Blast2GO [14]–[16]. The non-redundant (NR) database was searched using BLAST, with the *E*-value (expectation) set as 1e^−10^. Our results showed that Gene Ontology (GO) codes corresponding to 7 sequences were not annotated (Table S4). Gene Ontology (GO) categorization based on molecular function (MF), biological process (BP) and cellular component (CC) was done based on GO numbers of differentially expressed proteins using BLAST (see Table S5, Table S6, Table S7, Table S8, Figure S2, Figure S3, Figure S4, Figure S5, Figure S6 and Figure S7). The pie graphs were made according to the distribution of the target sequences (Figure 5A, Figure 5B, and Figure 5C). Binding activity and catalytic activity, among others, accounted for the largest proportion of all categories of MF (Figure 5A); metabolic process, cellular process, and response to stimulus accounted for the largest proportion of all categories of BP (Figure 5B); and the ratio of cells and organelles was the largest category in CC (Figure 5C). Based on this, a directed acyclic graph (DAG) containing MF, BP, and CC data was also made using the Blast2GO software (Figure 6 and Figure S8). A node with a score of 3.0 for receptor activity was found in the MF-DAG and confirmation that the receptor was HrBP1 was done using proteomic data. A subsequent node indicating signal transduction functions and a score of 1.8 was also observed in the MF-DAG (Figure 6). In addition, an initial signal transduction node with a score of 3.0 was found in the BP-DAG, and subsequent nodes represented signal transduction and regulation of cellular processes with node scores reaching 1.8 (Figure S8). These findings suggested to us that the antiviral effects of Dufulin are related to signal transduction and that the receptor protein HrBP1 could be its initial target due to the protein’s role in signal transduction. Finally, similar to the MF-DAG and BP-DAG results, the antiviral effects of Dufulin may be attributed to signal transduction triggered by HrBP1. In the analysis of CC-DAG, a node with a high score of 8.09 was found for “cell”, and the next node with score reaching 7.37 indicated “intracellular”; these results suggested that the antiviral responses are completed within the plant cell (Figure S9). Taken together, these data strongly suggest HrBP1 as a target protein of Dufulin and further confirmation should be done via molecular biology methods.

### Gene Expression Levels of Some Differentially Expressed Proteins

**Figure 7:**
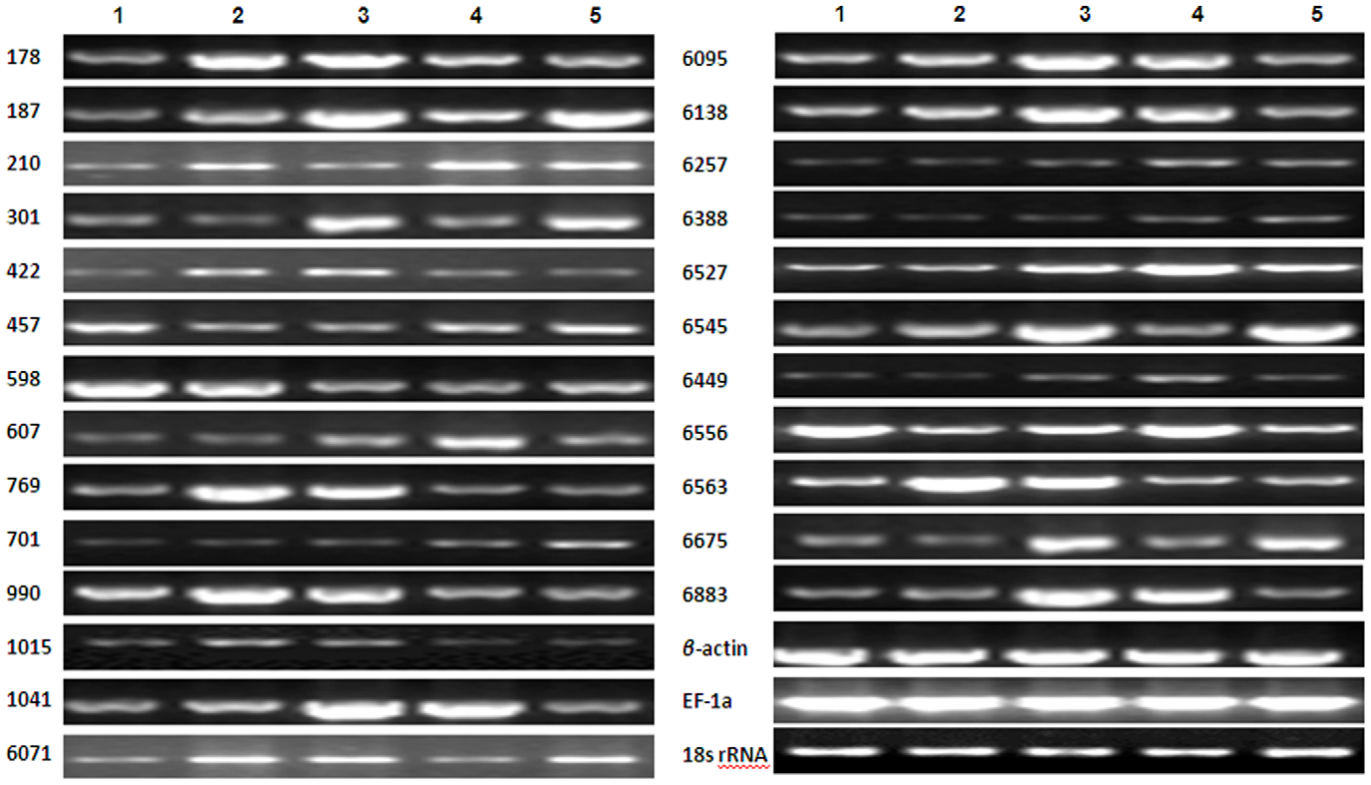
Gene expression analysis of differentially expressed proteins from the proteomics assay by semi-quantitative RT-PCR. Genes encoding the 26 different proteins listed in Table 5 were analyzed. Semi-quantitative RT-PCR was performed using gene-specific primers, and *β*-*actin*, *EF-1a* and *18S ribosomal RNA* genes were used as internal controls.

For many proteins that appeared to be upregulated in proteomic assays, expression at the mRNA level was also evaluated. Primers for these genes were designed using the Beacon Designer software (Table 4). Gene expression levels were examined using semi-quantitative RT-PCR (18 Cycles) and electrophoresis on 2.2% agarose gel. Many genes were found to be upregulated in tobacco leaves infected with TMV and subsequently treated with Dufulin. These changes in expression at the mRNA level detected by RT-PCR were in accord with changes at the protein level as indicated by DIGE and 2-DE analyses (Figure 7).

**Table 4:** 2D-DIGE experimental design.

Number of the gel	Cy2	Cy3/Cy5	Cy5/Cy3
1	Pool	A2	B3
2	Pool	B2	C3
3	Pool	C2	A4
4	Pool	B5	C4
5	Pool	B4	A5
6	Pool	C5	A3

Each gel was loaded with 50 µg of the Cy2-labeled protein pool from all samples (as an internal standard), 50 µg of the Cy3-labeled sample, and 50 µg of the Cy5-labeled sample, as indicated.

### Enhanced Expression of Crucial Genes and Proteins in the SA-mediated Signaling Pathway

**Figure 8:**
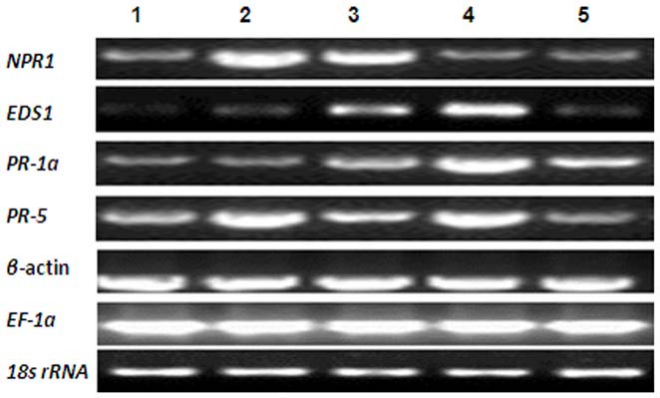
Gene expression analysis of crucial components of the SA signaling pathway by semi-quantitative RT-PCR. The genes encoding *EDS1*, *NPR1*, *PR-1a* and *PR-5* were analyzed. Semi-quantitative RT-PCR was performed using gene-specific primers, and *β-actin*, *EF-1a* and *18S ribosomal RNA* genes were selected as internal controls.

**Figure 9:**
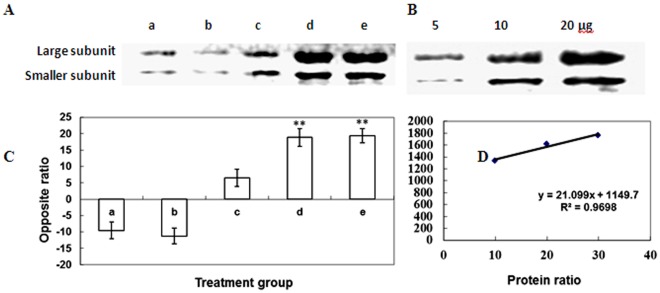
Protein expression analysis of plastid-lipid-associated proteins by western blot. (**A**). Semi-quantitative western blot of plastid-lipid-associated proteins. The letters a, b, c, d, and e represent the treatment groups described previously in “Experimental Procedures”. (**B**) Standard protein samples (5, 10 and 20 µg) were loaded on the well and analyzed by western blotting. (**C**) Optical density values obtained from the imaging software. The protein ratios were extrapolated using linear regression analysis and expressed as mean ± SD (n  = 4). Error bars represent standard deviation (SD). Asterisks indicate values significantly different from the mean at *P*<0.05. (**D**) Linear regression was used to quantify the differences between the bands in (**B**).

HrBP1 was identified as the first protein in the EDS/SA/NPR1/AOX/PR signaling pathway [1]. Activation of HrBP1 leads plants to exhibit antiviral activity by generating SAR [17], [18]. In addition, some molecules in downstream of the SA signaling pathway, such as the gene of NPR1, EDS1, lipid associated protein, PRs and phytoalexins, are regarded as markers of antiviral responses [19], [20]. Therefore, *NPR1, EDS1, PR-1a* and *PR-5* were examined as putative target genes in the activated state, and the degree of SA signaling and the level of SAR were evaluated [19]. Data obtained from semi-quantitative RT-PCR indicated that these genes were also upregulated after Dufulin was sprayed on the leaves of *N. tabacum* K_326_ (Figure 8), revealing that Dufulin effectively activates the SA signaling pathway and SAR. In addition, trend analysis of expression by western blotting indicated that the lipid associated protein was also upregulated by Dufulin (Figure 9).

### Induction of *PR-1a* Gene Expression by Dufulin and Other Inducers of SAR

**Figure 10:**
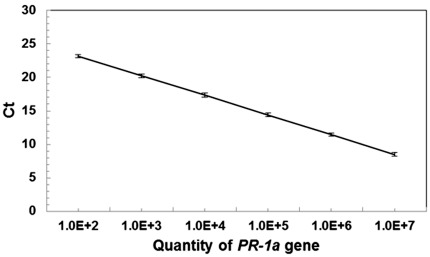
Standard curves for the *PR-1a* gene obtained in real-time PCR assays. Ct values represent the mean of 10 measurements and are shown on the *y*-axis, whereas differential concentrations are shown on the *x*-axis. Error bars represent standard deviation (SD).

**Figure 11:**
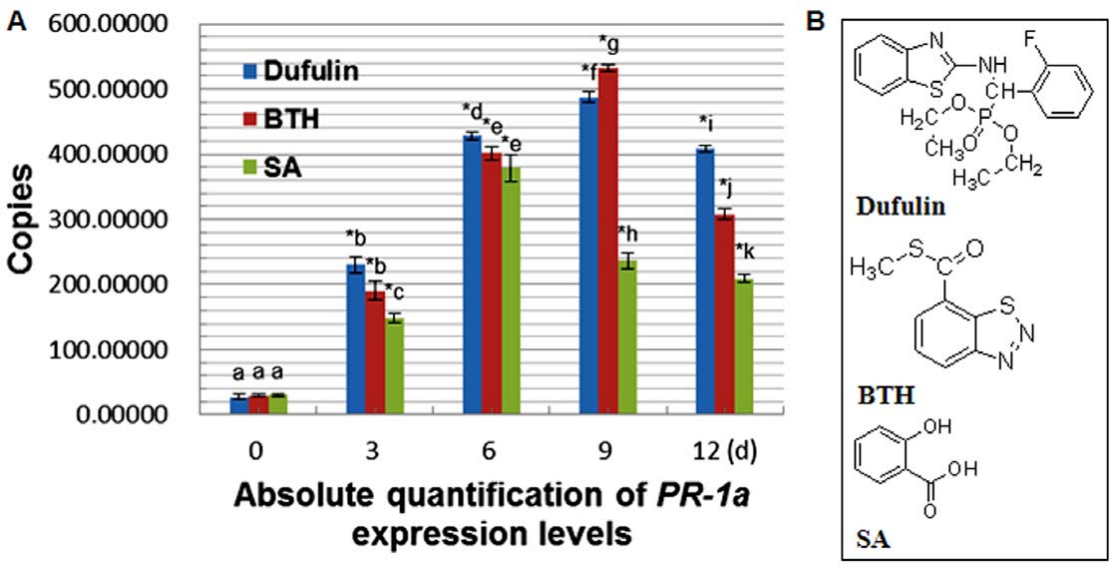
*PR-1a* gene expression analysis by qPCR. (**A**) Quantitative real-time PCR was performed using gene-specific primers (listed in Table 5) and the SYBR Green Realtime Master Mix. Expression ratios of the *PR-1a* gene in plants treated with BTH, Dufulin and exogenous SA at 500 µg/mL at 0, 3, 6, 9 and 12 days. Asterisks indicate statistically significant difference (P<0.05). The different lowercase letters indicate *PR-1a* expression values that are significantly different between the different treatment groups at the same treatment time at *P*<0.05. (**B**) Molecular structures of Dufulin, BTH and SA.

BTH and exogenous SA were known as inducers of SAR [21]–[23]. BTH was the first successful commercial plant activator, whose target of action has been shown to fall between *NPR1* and the SA molecules [21], [22]. Exogenous administration of SA on plants can increase the levels of SA, promote *NPR1* expression and ultimately increase the amount of PR protein [23]. In order to better understand the antiviral activity and application of Dufulin, we decided to compare the effect of Dufulin with that of BTH and exogenous SA on the expression level of *PR-1a.* The three compounds were sprayed onto the leaves of *N. tabacum* K_326_ at 500 µg/mL, respectively, and the expression status of *PR-1a* was determined at different time points by real time PCR (Figure 10 and Figure S10). Interestingly, expression of *PR-1a* was found to commence the fastest in the Dufulin treatment group, followed by the BTH and the exogenous SA treatment groups. Maximal expression of *PR-1a* occurred at 9, 9 and 6 days in the BTH, Dufulin and exogenous SA treatment groups, respectively. Over the course of 12 days, *PR-1a* expression was maintained the longest in the BTH treatment group, followed by the Dufulin treatment group and the exogenous SA treatment group (Figure 11).

**Figure 12:**
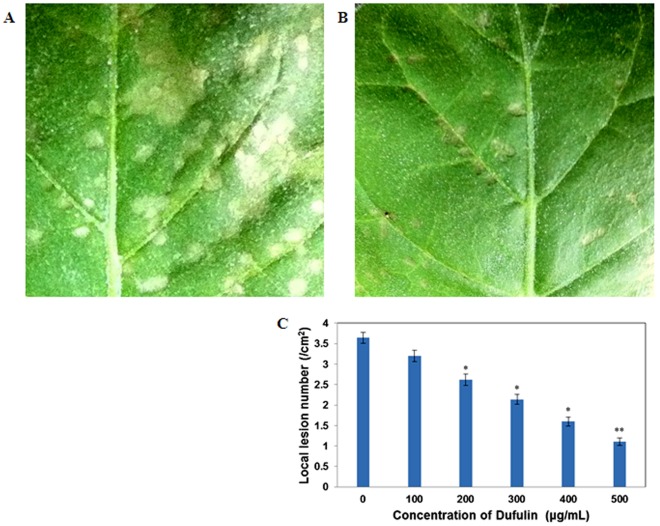
Dufulin-induced virus resistance in *N. glutinosa*. (**A**) Local lesions in the leaves of *N. glutinosa* after inoculation of 0.05% DMSO solution (the CK treatment group). (**B**) Local lesions in the leaves of *N. glutinosa* after inoculation with Dufulin at 500 µg/mL. Small white needle-like points evenly distributed on the leaves represent silicon carbide. (**C**) Number of local lesions. Values are expressed as the mean of at least 5 replicates. Asterisks indicate statistically significant differences at *P<0.05* (*) and *P<0.01* (**) when compared with the controls.

### Protective Activity of Dufulin Against TMV-induced Lesions

TMV, the most common pathogen in China affecting tobacco, tomato, cucumber, and potato, was selected for evaluation of antiviral bioactivity of Dufulin [24]. The ability of the antiviral agent to protect plants against local lesions caused by TMV was examined in our assays [25]. Varying concentrations of Dufulin were sprayed onto the tobacco leaves of 8-week-old *N. glutinosa* plants, and TMV inoculation was performed 5 days later. Dimethyl sulfoxide (DMSO; 0.05%) was used as the solvent control (the CK treatment group) (Figure 12A, Figure 12B). The average numbers of local lesions were 3.2±0.14, 2.6±0.14, 2.1±0.12, 1.6±0.11, and 1.1±0.09 lesions/cm^2^ for 100, 200, 300, 400, and 500 µg/mL administration of Dufulin, respectively (Figure 12A, Figure 12B). These values were significantly lower than those in the CK treatment group (3.6±0.13 lesions/cm^2^) (Figure 12C). Our results demonstrate that Dufulin inhibits lesions caused by TMV in a dose-dependent manner.

**Table 5:** Primer sequences used in semi-quantitative RT-PCR and qPCR.

Spot no.	GI	Gene name	Forward primer	Reverse primer	Product (bp)
178	U35619.1	bisphosphate carboxylase oxygenase	5′-gtgcaaacattttggcaatg-3′	5′-attcagcaactgcatctgga-3′	178
187	U15605.1	truncated N protein	5′-ggggagtcggtaaaacaaca-3′	5′-cagagagaagggcattttgc-3′	153
210	AJ866275.1	putative cell cycle protein	5′-tggtaccgggaacttcagtc-3′	5′-tggtcacagagctccatttg-3′	228
301	NC_001879	ATP synthase CF1 alpha subunit	5′-aaaagggcttctgcttcctc-3′	5′-aaaagggcttctgcttcctc-3′	153
422	Z14980.1	ribulose-bisphosphate carboxylase activase	5′-cggagaattggaaagtggaa-3′	5′-acgtttgtcgggttatcagc-3′	219
457	AJ250431.1	beta-galactosidase	5′-agcctggaactgattgtgct-3′	5′-tggttgggactttttcttgg-3′	240
598	gi|223593	carboxylase/oxygenase	5′-tcagatcaagggcattttcc-3′	5′-caagcgccatatcagcatta-3′	240
701	DQ442995	Phosphomannomutase	5′-agcacctcgcaaggaagtta-3′	5′-tccttgagcttctcgtctcc-3′	240
769	AY383625.1	harpin binding protein	5′-aatttgccagtcgggtattg-3′	5′-caatttccacctcccttgaa-3′	247
990	AB449366	germin like protein	5′-ggcttagtgcagctggaaac-3′	5′-cagggtgagtgtgaaatgga-3′	153
1015	AY220076.1	oxygen evolving complex photosystem II	5′-acctgcagttgaggtctgct-3′	5′-catttgctcctgagacgaca-3′	180
1041	gi|31711507	24 K germin-like protein	5′-cgatttctgtgttggggact-3′	5′-ggtgtgactgcggctttaat-3′	150
5942	gi|12643758	ribulose bisphosphate carboxylase/oxygenase-2(RuBisCO activase)	5′-tgttcctgctgaagacgttg-3′	5′-agttggtggtccgtcaaaag-3′	175
6071	J02979.1	lignin-forming peroxidase	5′-agcccttgcatctgaaattg-3′	5′-ttccaaatgtgtgtgcacct-3′	215
6095	gi|255542955	NAD-dependent epimerase/dehydratase	5′-gcaggcaaaaggagcactac-3′	5′-ggtctgattcacctggcaat-3′	179
6138	XM_002520879	stability/assembly factor HCF 136	5′-acatccaatgggggatacaa-3′	5′-cggcttgagacagcaacata-3′	164
6257	Y15489	plastid-lipid-associated protein	5′-agcagaggaagaaccaccaa-3′	5′-ctcggtaggagcaggagttg-3′	178
6288	AY220076.1	complex 33-kDa photosystem-II protein	5′-acctgcagttgaggtctgct-3′	5′-catttgctcctgagacgaca-3′	180
6388	AM087457.1	chloroplast cysteine synthase 1	5′-cttattgttgccgtggacct-3′	5′-gcaatgtttgcaacacaacc-3′	157
6449	DQ222496.1	cytosolic isoform of triosephosphate isomerase	5′-gttgtgagccctccatttgt-3′	5′-ccaaaattacccacggaatg-3′	163
6527	gi|52000814	oxygen-evolving enhancer protein 2-1	5′-gtagcgatcgccttcttgac-3′	5′-ttggggttaataggggaagg-3′	204
6545	AB120535	CDK-5 regulatory subunit associated	5′-gtcaagcaaaggaggctcac-3′	5′-atgacaccctatggcctctg-3′	169
6556	X64198	photosystem I light-harvesting chlorophylla/b-binding protein	5′-gtttccccctctgtcctctc-3′	5′-gggcagaaccatcaaggtaa-3′	151
6563	AB093097	Superoxide dismutase	5′-agctacatgacgccatttcc-3′	5′-ccctgtaaagcagcaccttc-3′	238
6675	D13718.1	PSI-D1 precursor	5′-ctcaaattggcccgtaaaga-3′	5′-tgaaattctgcccaactcct-3′	181
6883	AY220079.1	carboxylase small subunit precursor protein	5′-catggttgcacctttcactg-3′	5′-ttccaaacaaggaacccatc-3′	238
	AF480488	NPR1	5′-tagcgtattgcgatgcaaag-3′	5′-ttccatcggatgtcagatca-3′	180
	AF480489	EDS1	5′-aggccgaagcgttataggtt-3′	5′-aaaacatcatcgcccagaag-3′	203
	X05959.1	PR-1a	5′-caatacggcgaaaacctagctga -3′	5′- cctagcacatccaacacgaa-3′	170
	X03913	PR-5	5′-gcttccccttttatgccttc-3′	5′-cctgggttcacgttaatgct-3′	150
reference gene	GQ339768.1	*β*-actin	5′-ttgtccgtgacatgaaggag-3′	5′-atcatggatggctggaagag-3′	178
reference gene	AF120093.1	EF-1a	5′-ctctcaggctcccacttcag-3′	5′-aagagcttcgtggtgcatct-3′	164
reference gene	HQ384692.1	18S ribosomal RNA gene	5′-atggccgttcttagttggtg-3′	5′-tgtcggccaaggctataaac-3′	235

## Discussion

HrBP1 and the harpin-like binding protein are both found in plant cell walls and belong to a family of complex receptors. Activated HrBP1 can act upstream of the SA, JA, and ethylene (ETH) signaling pathways in plant cells [1]. Activation of HrBP1 can thus induce resistance against viruses, bacteria, fungi and pests in plants, and improve plant growth and development via those signaling pathways [5].

Here, we show that Dufulin can upregulate the expression of HrBP1 and activate the SA signaling pathway, leading to the upregulation of *PR-1a* and *PR-5*. Other studies have shown that the SA signaling pathway is closely associated with antiviral resistance of host plants and that its downstream molecules are PRs [21] and PAL protective enzymes [26], among which *PR-1a* and *PR-5* are closely related to antiviral resistance [27]. Earlier studies demonstrated that Dufulin can induce resistance in tobacco by activating PAL, peroxidase (POD), and superoxide dismutase (SOD). The results of the current study extend those findings by revealing that Dufulin induces antiviral resistance in the host plant by activating HrBP1 to trigger the SA signaling pathway, thereby producing SAR [9].

We further analyzed the activation of the SA signaling pathway by Dufulin and other known immunoactivators. We found that the time of induction of *PR-1a* expression after Dufulin treatment was quicker than that after exogenous SA application, and the maximal level of *PR-1a* gene expression in the Dufulin treatment group was higher than that in the exogenous SA treatment group. In addition, *PR-1a* gene expression persisted longer in the Dufulin treatment group than that in the SA and BTH treatment groups. Moreover, a full set of methods that combine proteome technology and bioinformatics was created to identify the target of the drug [28]–[30]. To our knowledge, this is the first example of a study combining aspects of modern biotechnology, namely proteomics and bioinformatics, and molecular biology to find a drug’s target of action against a plant viral disease.

In summary, this study used DIGE and 2-DE in combination with MS techniques and the Kyoto Encyclopedia of Genes and Genomes (KEGG) to identify HrBP1 as a possible target protein of Dufulin. Our result was verified by western blotting assays against lipid associated protein, and the downstream signaling pathway was identified by RT-PCR analysis of the related genes *NPR1*, *EDS1*, *PR-1a* and *PR-5*. All results obtained using our proteomics and molecular biology methods indicated that acquisition of SAR and enhancement of antiviral activity by Dufulin were mediated via HrBP1 activation. In addition, a protective assay based on counting of lesions also indicated that Dufulin had protective effects against TMV. Taken together, these results show that Dufulin is an immunoactivator of SAR that confers antiviral activity to cells via regulation of the SA signaling pathway. The findings of this research lay the groundwork for developing a molecular model for selection of antiviral drugs based on the SA signaling pathway.

## Materials and Methods

### Plant Materials and Growth Conditions

*Nicotiana tabacum* K-326 and *N. glutinosa* seeds were purchased from the Laboratory of Germplasm Genetic Resources of the Tobacco Research Institute, Chinese Academy of Agricultural Sciences. TMV was obtained from the Wuhan Institute of Virology at the Chinese Academy of Sciences, and its source was preserved in an *N. tabacum* K-326 plant over an extended period. The extraction and purification of TMV were carried out as previously described by Gooding [31]. *N. tabacum* K-326 and *N. glutinosa* were grown in a disease-free greenhouse, and 12-week-old plants were used for the study. The temperature and humidity levels in the greenhouse were maintained close to 25°C and 60%, respectively.

### Protein Sample Preparation for Proteomic Analysis

The leaf samples were divided into six groups: group A (control), healthy tobacco leaves; group B, tobacco leaves infected with TMV for 24 h; group C, tobacco leaves infected with TMV for 24 h and then treated with Dufulin for 24 h; group D, tobacco leaves infected with TMV and subsequently treated with Dufulin for 72 h; group E, tobacco leaves infected with TMV and then treated with Dufulin for 120 h; and group F, tobacco leaves infected with TMV for 120 h. The tobacco leaves were first washed with sterile water at room temperature and then inoculated with TMV. Powdered Dufulin was dissolved in an appropriate amount of dimethyl formamide (DMF) and mixed in phosphate-buffered saline (PBS) with 1% Tween-20 to obtain 500 mg/L solutions. The dried leaves were sprayed with Dufulin at a concentration of 500 mg/L at 24 h intervals. Samples of tobacco leaves were removed from the main leaf vein, frozen in liquid nitrogen, and stored at −80°C before use.

Total proteins were extracted from groups A–E using a modified trichloroacetic acid/acetone procedure as follows [10]–[12]. The samples were ground into a fine powder in liquid nitrogen, transferred into a tube containing 25 mL of trichloroacetic acid/acetone (1∶9, v/v) as well as 65 mM dithiothreitol, and then precipitated at –20°C for more than 1 h. The samples were centrifuged at 10,000 rpm at 4°C for 45 min, and the supernatants were discarded. Twenty-five milliliters of acetone was added to the precipitate, and the solutions were stored at −20°C for 1 h and centrifuged at 10,000 rpm at 4°C for 45 min, after which the supernatant was removed. The precipitate was vacuum-dried, weighed, and stored at −80°C. A total of 500 mg of dry powder samples was added to 500 µL of DIGE lysis buffer containing 7 M urea, 2 M thiourea, 4% CHAPS (3-[(3-cholamidopropyl) dimethylammonio]-1-propanesulfonate), and 0.2% immobilized pH gradient (IPG) buffer. The ratio of the volume of the lysis buffer and that of the enzyme inhibitors was maintained at 50∶1 and the mixture was stirred to homogeneity before spinning at 12,000 rpm at 4°C for 1 h. The supernatant was collected and filtered using a 0.22 µm filter to obtain a clear solution. The pH of the supernatant was adjusted to 8.0 for the DIGE experiment.

### 2D-DIGE and Image Analysis

According to the instructions provided with the Bio-Rad Protein Assay Kit (http://www.bio-rad.com/LifeScience/pdf/Bulletin_9004.pdf), the concentrations of all the samples were adjusted to 5 µg/µL. An internal standard was prepared by mixing equal amounts of all samples to eliminate systemic and inherent biological variations. The samples and internal standards were labeled with DIGE dyes for 30 min in an ice bath under dark conditions using 50 µg of protein and 400 pmol of fluorescent dyes. The reaction was terminated by adding 1 µL of 10 mM lysine after 10 min. The internal standard was labeled with Cy2, half the number of samples from each group was labeled with Cy3, and the remaining half was labeled with Cy5. The multiplexing of all samples was also randomized separately to keep the results unbiased, as shown in Table 4. IPG strips (pH 3–10, 13 cm) (http://www.gelifesciences.com/) were cup-loaded with 50 µg of protein of each of the Cy2-, Cy3-, and Cy5-labeled samples in a buffer containing 7 M urea, 2 M thiourea, 2% (w/v) CHAPS, 65 mM dithiothreitol, and 1% (v/v) IPG buffer. Isoelectric focusing was carried out under the following conditions: 12 h at 30 v, 1 h at 500 v, 1 h at 1000 v, 8 h at 8000 v, and 4 h at 500 v. Sodium dodecyl sulfate–polyacrylamide gel electrophoresis (SDS-PAGE) was carried out on a 12.5% gel using Hoefer SE600 (Hoefer-GE Healthcare Apparatuses). Second-dimension SDS-PAGE was run by overlaying the strips on 12.5% isocratic Laemmli gel (13×13 cm) cast in low-fluorescence glass plates on an Ettan DALT VI system. Gels were run at 20°C at a constant power of 2.5 W/gel for 30 min, followed by constant power of 17 W/gel until the bromophenol blue tracking front ran off the gel. Fluorescence images of the gels were acquired on a Typhoon 9400 scanner. Cy2, Cy3, and Cy5 images for each gel were scanned at excitation/emission wavelengths of 488/520, 532/580, and 633/670 nm, respectively, at 100 µm resolution, yielding 27 images. Protein spots in both the analytical and preparative gels were observed using the silver staining procedure as described previously [13]. Spot identification, background elimination, point matching, and differential analysis of the protein spots were completed using a 2D DeCyder software (Differential In-Gel Analysis and Biological Variance Analysis Version 4.0; Amersham Biosciences, Piscataway, NJ). The biological variation analysis module was used for inter-gel matching of the internal standard, and all the gel samples were subjected to comparative cross-gel statistical analysis based on spot volumes with a t-test. *P* value <0.01 was considered statistically significant. Importantly, spots corresponding to the same protein were identified in different gels and their trends of up-regulation and down-regulation were validated in all the gels in order to avoid false positives. At least three sets of gels were run for each sample.

### 2-DE and Image Analysis

Although the DIGE experiments exhibited high sensitivity and good reproducibility, it was not effective in identifying low-abundance proteins as the loading volume in DIGE was significantly lower than that in traditional 2-DE. The total loading sample size was 150 µg for 3 samples labeled with Cy2, Cy3 and Cy5 in the DIGE experiments, but the total loading sample size was 800 µg for each sample in 2-DE. The traditional 2-DE test was introduced to overcome this shortcoming of lower loading volume in DIGE. Expression profiles were acquired by comparing the results between groups B and E, and results were obtained using both 2-DE and DIGE. For the comparative analysis, samples from these groups were first separated by 2-DE according to the conventional method [13], [32]. The gel was then stained via the silver staining method to identify protein spots with differential expression. The protein spots found by silver staining were excised from another SDS-PAGE gels dyed with coomassie brilliant blue, then identified by MS. Image analysis was done using the DeCyder differential analysis software (Version 4.0, Amersham Biosciences).

### Protein Digestion and Protein Identification by MS

Protein spots were excised from several gels stained with coomassie brilliant blue, destained in 30 mM potassium ferricyanide/100 mM sodium thiosulfate (1∶1, v/v) for 20 min, and subsequently with Milli-Q water. The spots were incubated in 0.2 M NH_4_HCO_3_ for 20 min and then lyophilized. Each spot was digested overnight in 2 µL of 12.5 ng/µL trypsin in 0.1 M NH_4_HCO_3_. The peptides were extracted thrice with a mixture of 50% acetonitrile and 0.1% trifluoroacetic acid. The digests of the main protein spots were analyzed on a MALDI-TOF/TOF mass spectrometer (AB SCIEX 4700 Proteomics Analyzer, USA) and a Finnigan linear ion trap quadrupole mass spectrometer (LTQ; ThermoQuest, San Jose, CA, USA). Tryptic digests were prepared according to the manufacturer’s instructions (AB SCIEX 4700 Proteomics Analyzer, USA). Tandem MS (MS/MS) data were acquired with a nitrogen laser at a sampling rate of 25 Hz. The data obtained were submitted to the MASCOT software for protein identification. The NR Protein Database (6,122,577 sequences; 209,623,0148 residues) of the National Center for Biotechnology Information (NCBI-nr 20080221) was then searched with reference to green plants. The other parameters involved in the search were trypsin, one missed cleavage, fixed modifications of the carbamidomethyl (C) group, and variable modifications in terms of oxidation (of Met). A peptide tolerance level of 100 ppm, a fragment mass tolerance of ±0.5 Da, and a peptide charge of 1^+^ were selected for the study. Only significant hits, as defined by the MASCOT probability analysis (*P*<0.05), were accepted.

Thirty-one protein spots were identified using the AB SCIEX 4700 Proteomics Analyzer, which was operated in reflection mode and calibrated using AB SCIEX 4700 Calibration Mixture (Applied Biosystems, Foster City, CA). The data obtained from MALDI-TOF/TOF MS/MS was analyzed by GPS Explorer software (version 2.0, Applied Biosystems). Both MS and MS/MS data were acquired with a neodymium:yttrium–aluminum–garnet laser at a sampling rate of 200 Hz. The MS/MS mode was operated at 1 kV of collision energy. Collision-induced dissociation was carried out using air as the collision gas. For MS spectra, the peaks were calibrated using trypsin auto-digestion peaks and subsequently smoothed. The signal-to-noise criterion was set at 25 or greater. Monoisotopic masses were processed for identification. For MS/MS spectra, the peaks were calibrated by default and smoothed. All the peaks were de-isotoped.

Four additional protein spots were identified using the Finnigan LTQ mass spectrometer (ThermoQuest) coupled with a Surveyor HPLC system (ThermoQuest). Reversed-phase HPLC was conducted using a Surveyor LC system (Thermo Finnigan, San Jose, CA) on a C_18_ column (RP, 180 µm×150 mm; BioBasic® C_18_, 5 µm, Thermo Hypersil-Keystone). The pump flow rate was divided 1∶120 to achieve a column flow rate of 1.5 µL/min. Mobile phase A consisted of 0.1% formic acid in water, whereas mobile phase B consisted of 0.1% formic acid in acetonitrile. The tryptic peptide mixtures were eluted using a gradient of 2%–98% B over 60 min. The temperature of the heated capillary was set at 170°C. A voltage of 3.4 kV applied to the electrospray ionization needle resulted in a distinct signal. The normalized collision energy was 35.0. The mass spectrometer was set up in such a way that one full scan was followed by three MS/MS scans on the three most intense ions in the spectrum. The Dynamic Exclusion™ settings were as follows: repeat count, 2; repeat duration, 0.5 min; and exclusion duration, 2.0 min.

### Bioinformatic Analysis

For the overall analysis of the data generated from the proteomic studies and the rapid identification of the target, a novel bioinformatics-based approach was used. The functional categories of the proteins were defined according to the GO database. The sequences were downloaded from GenBank based on their genInfo identifier (GI) number, and 13 redundant data were discarded. Furthermore, the sequence information for GI entries 76869447 and 51964984 were not acquired. The sequences were analyzed using the Blast2GO procedure [14]–[16], and the NR database was searched using BLAST with the *E*-value fixed at 1e^−10^. The GO numbers corresponding to the sequences were acquired through the abovementioned method, and the categorization (MF, BP and CC) of differentially expressed proteins was done based on their GO numbers using the BLAST software.

### Western Blotting Analysis

Leaves of *N. tabacum* K-326 plants were ground to a fine powder in liquid nitrogen and homogenized in an ice-cold mortar with homogenization buffer (200 mM NaCl, 1 mM EDTA, 0.2% Triton X-100, and 100 mM Tris–HCl, pH 7.8) supplemented with Complete Protease Inhibitor Cocktail (Roche, Basel) with 4% *β*-mercaptoethanol (2-ME). The homogenate was centrifuged at 12,000 g for 10 min at 4°C. The supernatant containing the target protein was measured and determined using the Bradford assay. Extracted proteins of the same amount in each treatment group were analyzed by SDS-PAGE followed by western blotting. Proteins were electrotransferred to a Hybond-P membrane (Amersham 13 Biosciences, Little Chalfont, UK) and detected using antibodies against lipid-associated proteins (1∶1500) as well as anti-rabbit IgG (alkaline phosphatase conjugate) antibody raised in goat (1∶3000; SIGMA). Antibodies were diluted in 5% non-fat milk in TBS-T buffer. The color reaction was terminated by adding nitroblue tetrazolium (NBT) (50 mg/mL) and 5-bromo-4-chloro-3-indolyl phosphate (BCIP) (50 mg/mL), and the blotting membrane was analyzed using Bio-Rad GelDoc XR [33], [34].

### RNA Extraction and RT-PCR

Tobacco leaf samples were collected and frozen immediately in liquid nitrogen until use. Total RNA was extracted using the Takara Trizol Reagent Kit. DNase digestion followed by passing through adsorption columns was carried out to eliminate possible contamination with DNA. Two micrograms of total RNA was used as a template to synthesize first-strand cDNA with SuperScript VILO cDNA Synthesis Kit (Life Technologies). The primers for 26 differentially expressed genes (based on proteomics), *EDS1*, *NPR1*, *PR-1a*, *PR-5*, *β-actin*, *EF-1a* and *18S ribosomal RNA* genes were designed with the Beacon Designer software using the gene sequences obtained from GenBank (Table 5). The primers were synthesized (Takara) and plasmid transformation and cloning were conducted using a pGEM-T Cloning Kit (Tiangen Biotech, Beijing, China). Plasmid standards were diluted 10-fold and calculated according to the following formulas:

1. Plasmid Sample (ng/µL)  =  OD_260_×50-fold Dilution.
2. Sample MW  =  Base Number×324.
3. Copies of Test Sample (copies/µL)  =  Concentration of Test Sample/Sample MW×6×10^14^.

The copies of unknown samples (copies/µL) were calculated using a regression equation. Quantitative real-time PCR was performed using the SYBR Green reagent mix in an iCycler iQ™ instrument according to the manufacturer’s instructions (Bio-Rad, USA). Semi-quantitative RT-PCR was also performed on a Bio-Rad iCycler iQ™ Real-Time PCR. qPCRs were run with a program consisting of 95°C denaturation for 30 second, followed by 58°C annealing for 30 second, and 72°C extension for 40 second for 40 cycles.

### Induction of Protective Activity Against TMV

Dufulin was dissolved in 0.05% DMSO to concentrations of 100, 200, 300, 400, and 500 µg/mL, and the solution was sprayed onto 8-week-old *N. glutinosa* plant leaves. Tobacco leaves that were sprayed with standard 0.05% DMSO solution comprised the control group. Five days after spraying, the plants were inoculated with TMV, and then maintained in a growth chamber at 25°C and 80% humidity under a 16-h light/8-h dark cycle for 3 days. The numbers of local lesions and of mottled leaves were documented. Five leaves of each plant were inoculated with TMV in each treatment group of at least four replicates [25]. The experiment was repeated thrice with similar results.

### Statistical Analysis

ANOVA was conducted using the statistical software SPSS (version 11.5). Means (of opposite ratios in western blotting assays, of Ct values and gene copies in RT-PCR analysis, and of lesion number in the protective assay) were compared using the LSD test (homogeneity of variance, P>0.05) or Dunnett’s T3 test (heterogeneity of variance, P<0.05).

### Supporting Information

Additional supporting information may be found in the online version of this article.

## Supporting Information

Figure S1 **The MS spectra for protein spots with different expression.** Part 1.The MS spectra for protein spots no.301. Part 2.The MS spectra for protein spots no.178. Part 3.The MS spectra for protein spots no.457. Part 4.The MS spectra for protein spots no.598. Part 5.The MS spectra for protein spots no.769. Part 6.The MS spectra for protein spots no.990. Part 7.The MS spectra for protein spots no.1015. Part 8.The MS spectra for protein spots no.1041. Part 9.The MS spectra for protein spots no.187. Part 10.The MS spectra for protein spots no.210. Part 11.The MS spectra for protein spots no.422. Part 12.The MS spectra for protein spots no.701. Part 13.The MS spectra for protein spots no.1042. Part 14.The MS spectra for protein spots no.1413-1. Part 15.The MS spectra for protein spots no.1413-2. Part 16.The MS spectra for protein spots no.6388. Part 17.The MS spectra for protein spots no.6675. Part 18.The MS spectra for protein spots no.6818. Part 19.The MS spectra for protein spots no.5942. Part 20.The MS spectra for protein spots no.6071. Part 21.The MS spectra for protein spots no.6138. Part 22.The MS spectra for protein spots no.6087. Part 23.The MS spectra for protein spots no.6095. Part 24.The MS spectra for protein spots no.6257. Part 25.The MS spectra for protein spots no.6288. Part 26.The MS spectra for protein spots no.6265. Part 27.The MS spectra for protein spots no.6287. Part 28.The MS spectra for protein spots no.6246. Part 29.The MS spectra for protein spots no.6291. Part 30.The MS spectra for protein spots no.6527. Part 31.The MS spectra for protein spots no.6563. Part 32.The MS spectra for protein spots no.6545. Part 33.The MS spectra for protein spots no.6449. Part 34.The MS spectra for protein spots no.6556. Part 35.The MS spectra for protein spots no.6395. Part 36.The MS spectra for protein spots no.6883.
(ZIP)

Figure S2 **Sequence distribution of molecular function (MF).**
(TIF)

Figure S3 **Sequence distribution of biological processes (BP).**
(TIF)

Figure S4 **Sequence distribution of cellular component (CC).**
(TIF)

Figure S5 **Direct GO count of MF.**
(TIF)

Figure S6 **Direct GO count of BP.**
(TIF)

Figure S7 **Direct GO count of CC.**
(TIF)

Figure S8 **DAG of BP.**
(TIF)

Figure S9 **DAG of CC.**
(TIF)

Figure S10 **Absolute quantity of the** ***PR-1a*** **gene. (A)** Log view of the amplification curve of *PR-1a* in the control group. **(B)** Log view of the amplification curves of *PR-1a* in the treatment groups. **(C)** Melt curve of *PR-1a* in the treatment groups. **(D)** Results of PCR and agarose gel electrophoresis of *PR-1a*.
(TIF)

Table S1 **Differentially expressed proteins identified by DIGE and MS.**
(DOCX)

Table S2 **The ratio of up-regulation to differentially expressed protein in group E comparing with that in group B in 2-DE.**
(DOCX)

Table S3 **Differentially expressed proteins identified by 2-DE and MS.**
(DOCX)

Table S4 **Sequence data (FASTA) of the differentially expressed proteins obtained from MS.**
(DOCX)

Table S5 **Gene Ontology (GO) categorization of differentially expressed protein spots based on their molecular function.**
(DOCX)

Table S6 **GO categorization of differentially expressed proteins based on their localization in various cellular components.**
(DOCX)

Table S7 **GO categorization of differentially expressed proteins based on their involvement in various biological processes.**
(DOCX)

Table S8 **KEGG categorization of differentially expressed proteins based on MS data.**
(DOCX)
