# Supplementary figures and images for: Dufulin Activates HrBP1 to Produce Antiviral Responses in Tobacco
Source: PLoS One. 2012 May 25;7(5):e37944. doi: 10.1371/journal.pone.0037944 (PMC3360678; doi:10.1371/journal.pone.0037944)

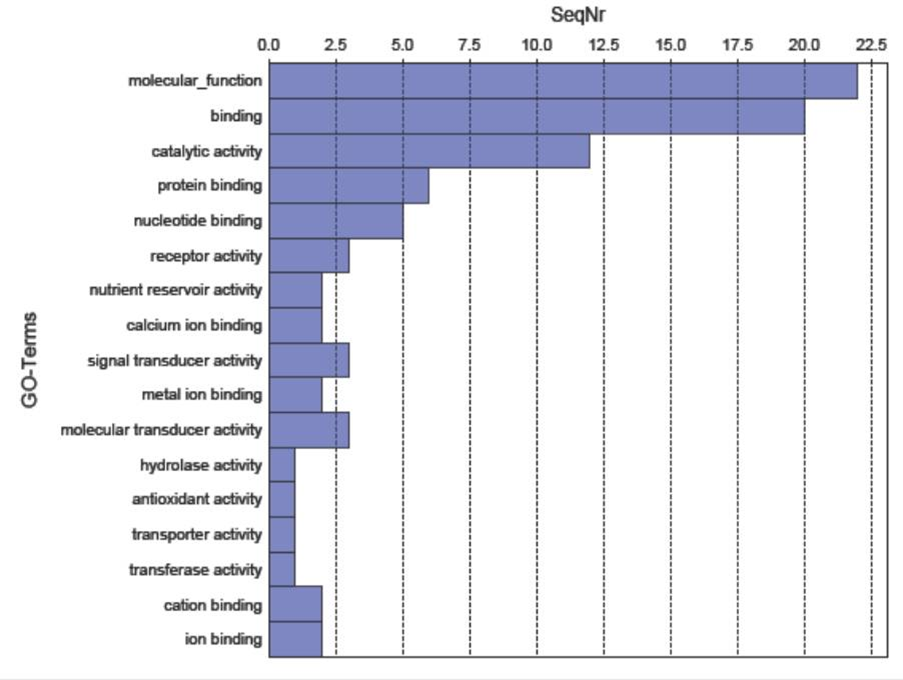

Supplement: Figure S2 — Sequence distribution of molecular function (MF). (TIF) [file pone.0037944.s002.tif]

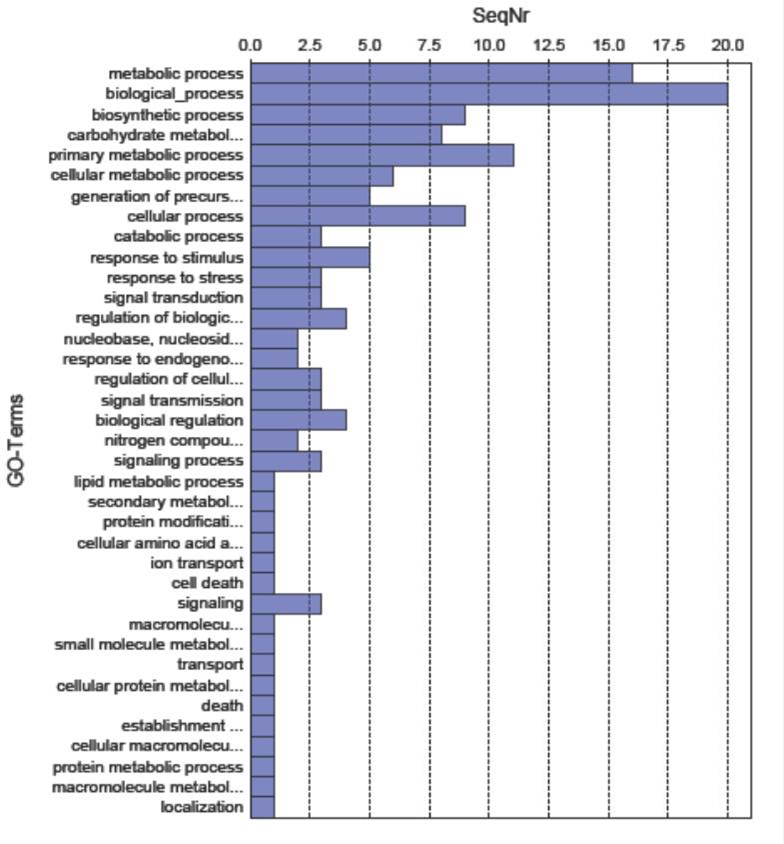

Supplement: Figure S3 — Sequence distribution of biological processes (BP). (TIF) [file pone.0037944.s003.tif]

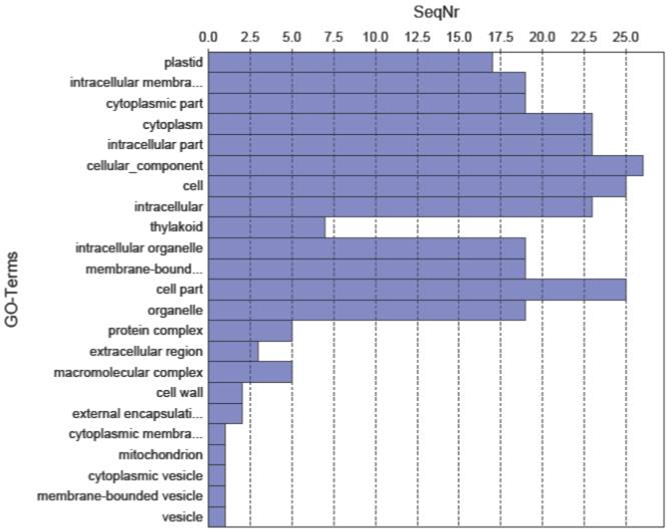

Supplement: Figure S4 — Sequence distribution of cellular component (CC). (TIF) [file pone.0037944.s004.tif]

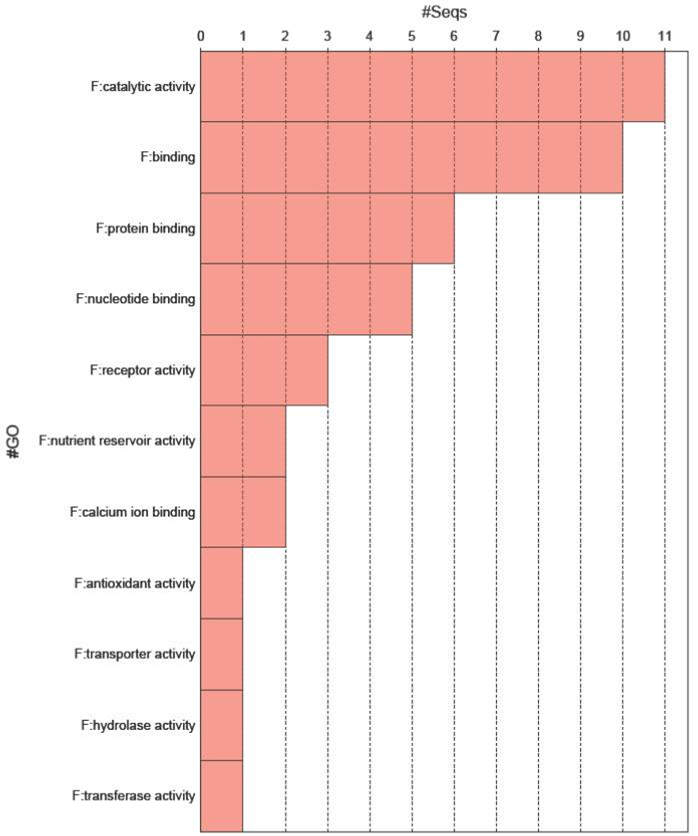

Supplement: Figure S5 — Direct GO count of MF. (TIF) [file pone.0037944.s005.tif]

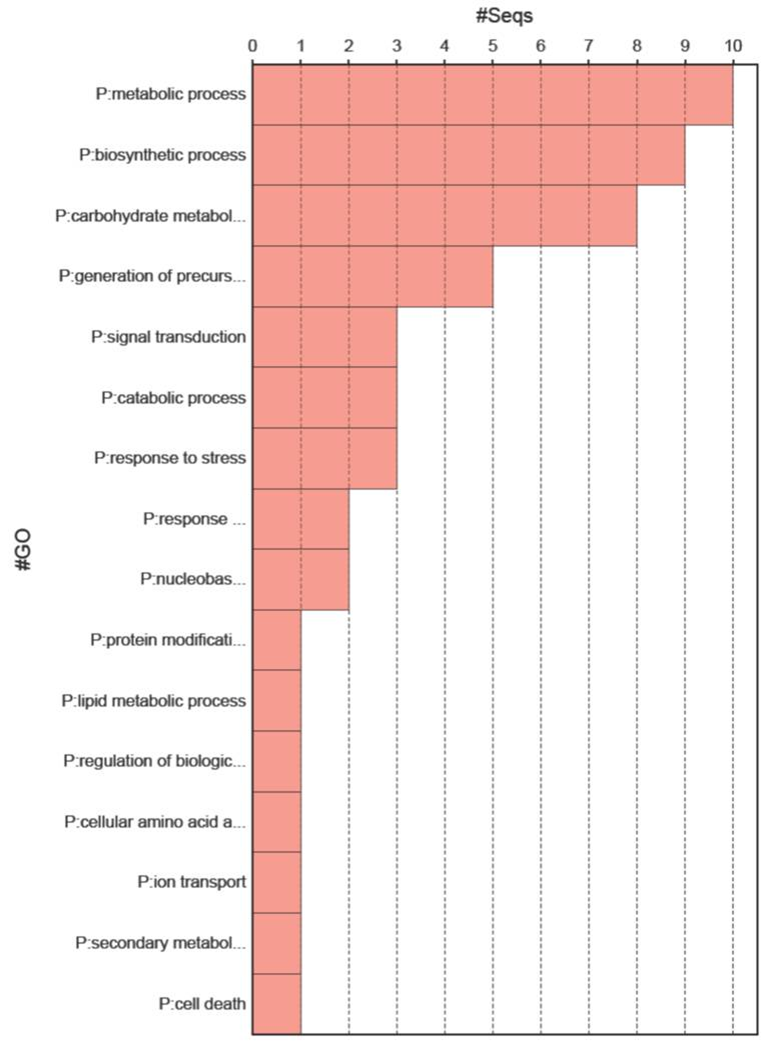

Supplement: Figure S6 — Direct GO count of BP. (TIF) [file pone.0037944.s006.tif]

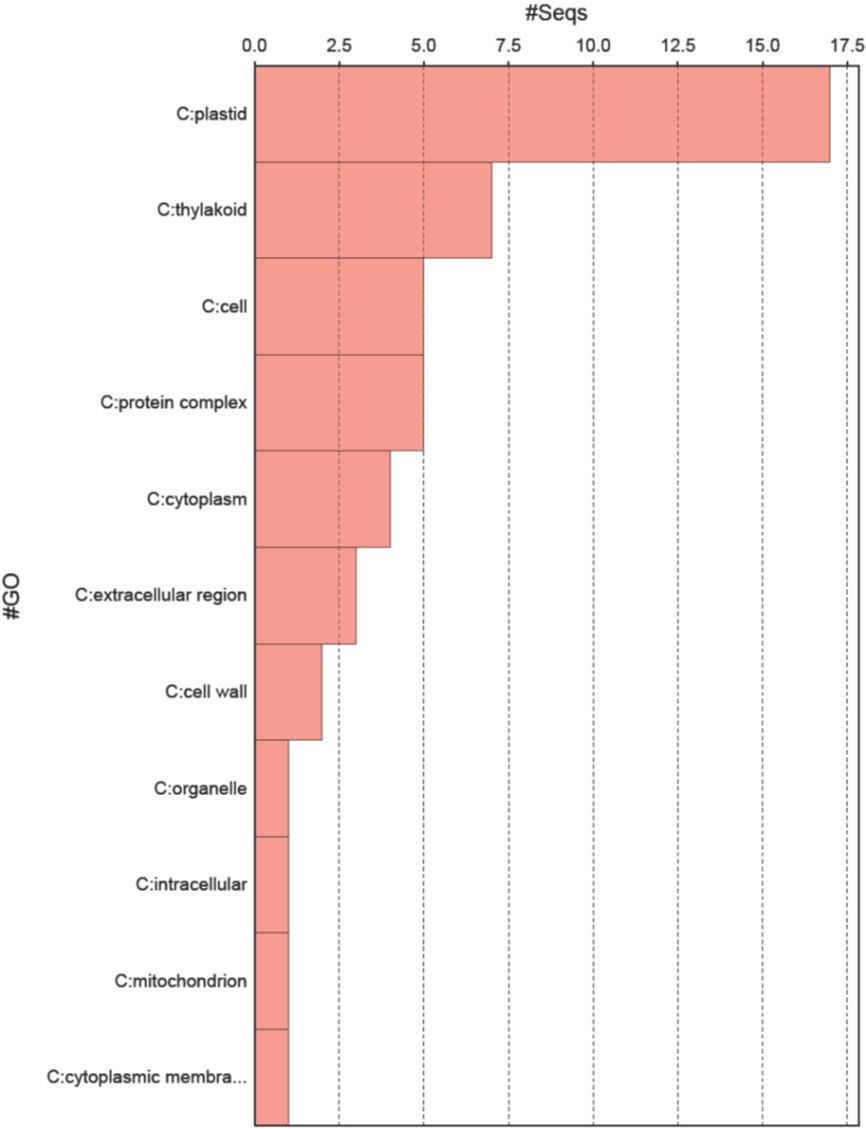

Supplement: Figure S7 — Direct GO count of CC. (TIF) [file pone.0037944.s007.tif]

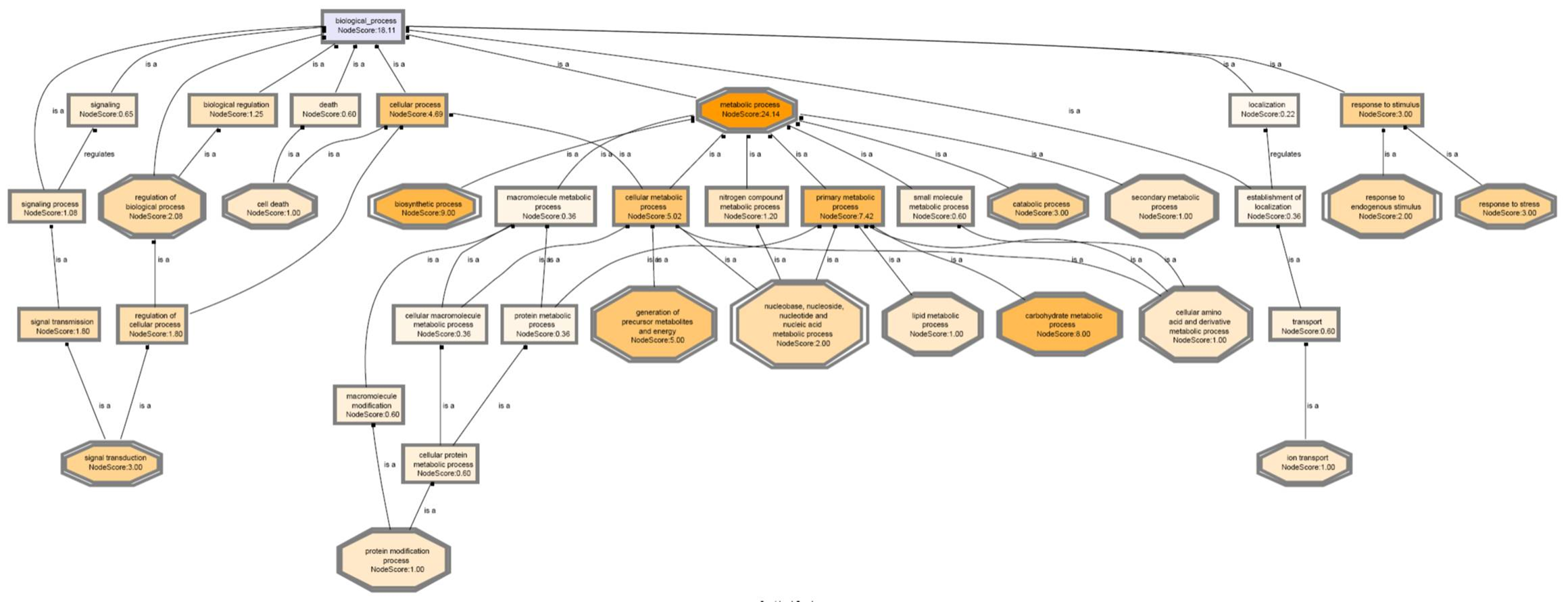

Supplement: Figure S8 — DAG of BP. (TIF) [file pone.0037944.s008.tif]

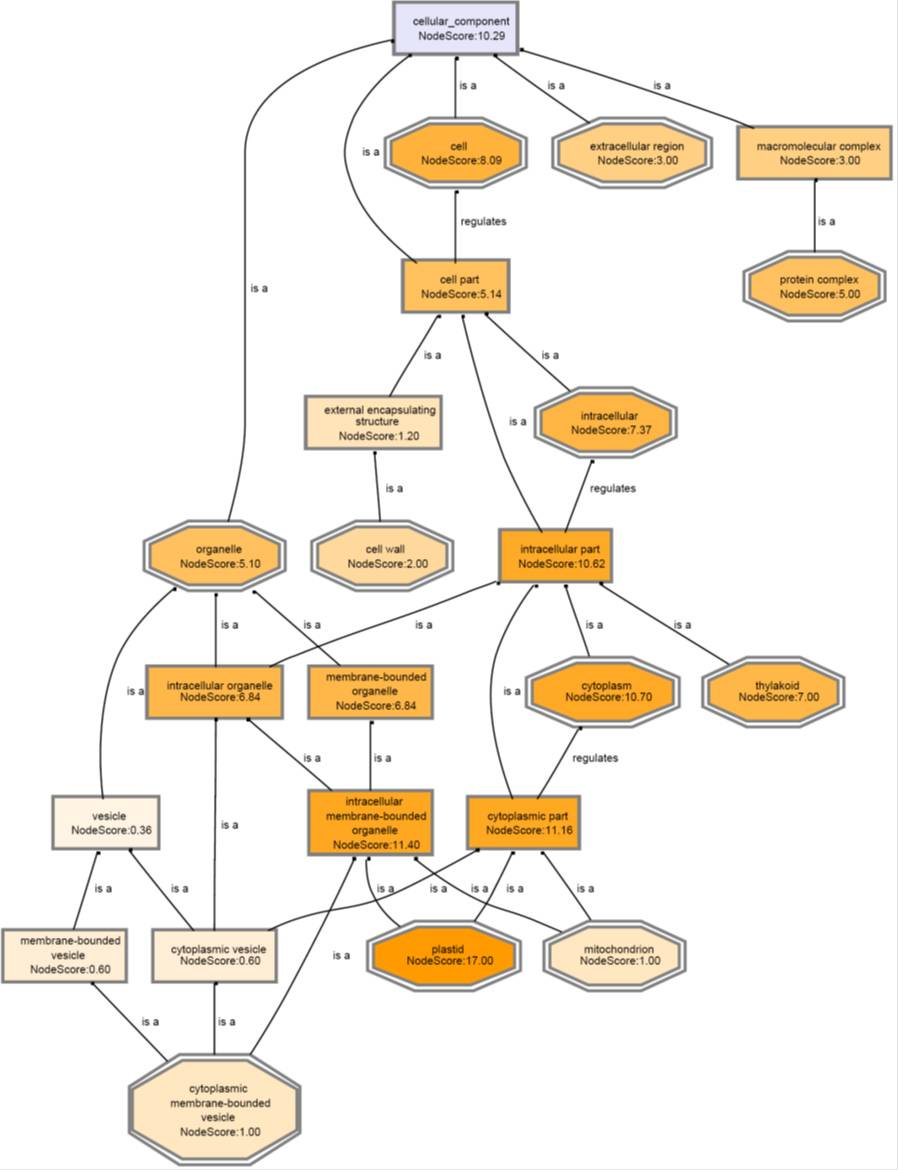

Supplement: Figure S9 — DAG of CC. (TIF) [file pone.0037944.s009.tif]

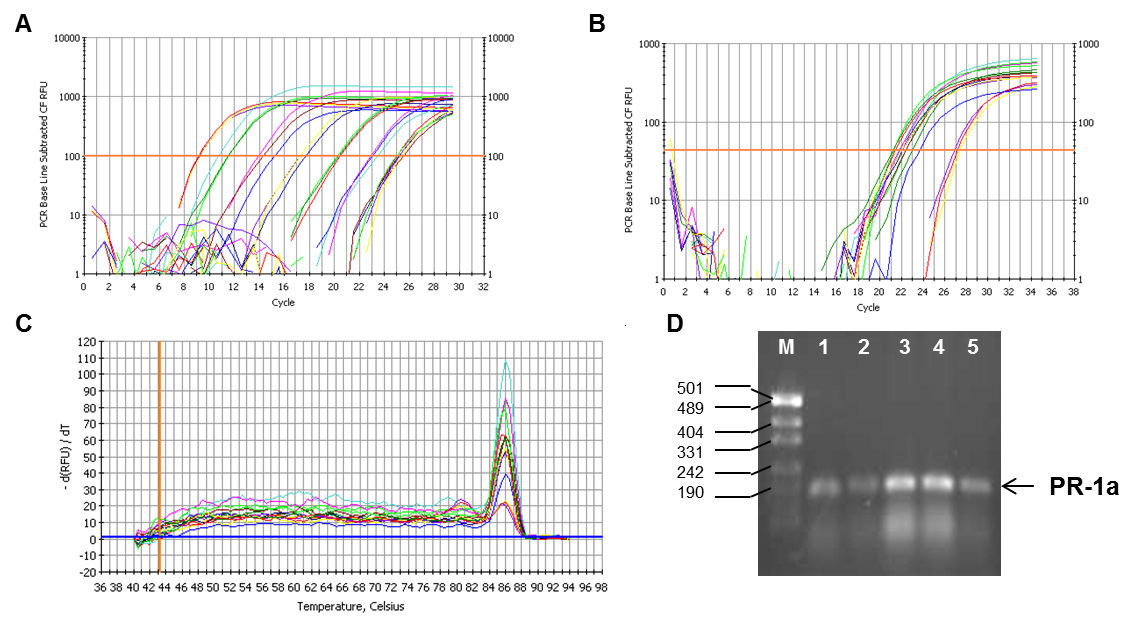

Supplement: Figure S10 — Absolute quantity of the PR-1a gene. (A) Log view of the amplification curve of PR-1a in the control group. (B) Log view of the amplification curves of PR-1a in the treatment groups. (C) Melt curve of PR-1a in the treatment groups. (D) Results of PCR and agarose gel electrophoresis of PR-1a. (TIF) [file pone.0037944.s010.tif]
