# Supplementary material for: Dufulin Activates HrBP1 to Produce Antiviral Responses in Tobacco
Source: PLoS One. 2012 May 25;7(5):e37944. doi: 10.1371/journal.pone.0037944 (PMC3360678; doi:10.1371/journal.pone.0037944)
Supplement: Table S1 — Differentially expressed proteins identified by DIGE and MS. (DOCX) [file pone.0037944.s011.docx]

**Table S1**

| **Protein Description** | | | | **Species** | | **GI Number** | | **Function Category** | | | **Subcellular Location** | | | **Theoretical MM (Da)** | | **pI** | | **Experimental MM (kDa)/pI** | | **Peptide count** | | **Protein Score/Expect** | | **Protein Score C.I %/Matches** | | | **Best Ion Score** | **One-Way ANOVA** |
| --- | --- | --- | --- | --- | --- | --- | --- | --- | --- | --- | --- | --- | --- | --- | --- | --- | --- | --- | --- | --- | --- | --- | --- | --- | --- | --- | --- | --- |
| **Proteins identified by MALDI-TOF/TOF Spectrometry (ABI 4700 Proteomics Analyzer, US)** | | | | | | | | | | | | | | | | | | | | | | | | | | | | |
| ATP synthase CF1 alpha subunit | | | | Nicotiana sylvestris | | gi\|78102516 | | energy metabolism | | | | mitochondrial membranes | | 55388 | | 5.14 | | 62.4/5.15 | | 19 | | 686 | | 100 | | | 136 | 0.014 |
| Chain A Of ribulose-1,5-bisphate carboxylase oxygenase | | | | Nicotiana tabacum | | gi\|515239 | | photosynthesis and photorespiration | | | | chloroplast membrane | | 49526.1 | | 6.19 | | 70.1/6.57 | | 14 | | 250 | | 100 | | | 56 | 0.039 |
| putative beta-galactosidase | | | | Solanum lycopersicum | | gi\|7939623 | | carbon metabolism | | | | lysosome | | 93183.9 | | 6.81 | | 46.9/7.28 | | 11 | | 339 | | 100 | | | 77 | 0.0082 |
| carboxylase/oxygenase, | | | | Nicotiana tabacum | | gi\|223593 | | carbon metabolism | | | | cytoplasm | | 14490.2 | | 4.99 | | 40.5/5.05 | | 5 | | 65 | | 78.524 | | | 29 | 0.026 |
| harpin binding protein 1 | | | | Nicotiana tabacum | | gi\|38679323 | | disease resistance | | | | cell membrane | | 29980 | | 8.8 | | 34.4/4.66 | | 8 | | 242 | | 100 | | | 72 | 9.8e-005 |
| germin like protein | | | | Nicotiana tabacum | | gi\|222051768 | | disease resistance | | | | cytoplasm | | 21408.2 | | 5.84 | | 28.2/5.77 | | 4 | | 257 | | 100 | | | 153 | 0.035 |
| oxygen evolving complex 33 kDa photosystem II | | | | Nicotiana tabacum | | gi\|30013657 | | photosynthesis and photorespiration | | | | chloroplast | | 35176.9 | | 5.63 | | 27.9/6.17 | | 11 | | 284 | | 100 | | | 78 | 0.00073 |
| 24K germin like protein | | | | Nicotiana tabacum | | gi\|31711507 | | disease resistance | | | | cytoplasm | | 21954.4 | | 7.82 | | 27.1/7.78 | | 4 | | 350 | | 100 | | | 178 | 0.046 |
| truncated N protein | | | | Nicotiana tabacum | | gi\|45544515 | | resistance disease | | | | cytoplasm | | 74429 | | / | | 70.15/5.53 | | 30 | | 4.9e+02 | | 4 | | | / | 0.029 |
| putative cell cycle protein | | | | Nicotiana tabacum | | gi\|82775180 | | cell division | | | | nucleus | | 20523 | | / | | 68.55/7.28 | | 22 | | 2.8e+03 | | 2 | | | / | 0.015 |
| **Proteins identified by LTQ Spectrometry (Thermo Finnigan, San Jose, CA)** | | | | | | | | | | | | | | | | | | | | | | | | | | | | |
| **Protein description** | **Species** | **GI no.** | **Sequence** | | **Function Category** | | **Subcellular location** | | **CoverPercent** | **Theoretical MM (Da)** | | | **PI** | | **Experimental MM (kDa)/pI** | | **MH+** | | **Diff(MH+)** | | **Charge** | | **Rank** | | **XC** | **DeltaCn** | | **One Way ANOVA** |
| 24K germin like protein | Nicotiana tabacum | gi\|31711507 | K.LNPLIK.A | | defense resistance | | cytoplasm | | 2.86% | 21968.49 | | | 5.83 | | 27.10/7.86 | | 697.889 | | 0.18904 | | 1 | | 1 | | 2.1532 | 0.3057 | | 0.0033 |
| phosphomannomutase | Nicotiana tabacum | gi\|90762161 | R.SGM*LNVSPIGR.D | | carbon metabolism | | cytoplasm | | 4.37% | 8575.432 | | | 5.71 | | 39.38/6.50 | | 1147.331 | | -0.93531 | | 2 | | 1 | | 2.4934 | 0.5942 | | 0.016 |
| ribulose-bisphosphate carboxylase activase | Nicotiana tabacum | **gi\|19992\|** | R.TDNVPEEAVVK.I  R.TDNVPEEAVVK.I  R.VYDDEVRK.W  R.VYDDEVRK.W | | photosynthesis and photorespiration | | chloroplast | | 8.19% | 25929.41 | | | 5.01 | |  | | 1201.309  1201.309  1024.109  1024.109 | | 0.39451  0.48751  -0.41359  0.18941 | | 2  2  2  2 | | 1  1  1  1 | | 2.4651  2.4786  2.6064  2.2002 | 0.6007  0.5422  0.5159  0.4997 | | 0.047 |
| ribulose bisphosphate carboxylase small subunit precursor | Nicotiana tabacum | gi\|30013663\| | R.GFVYRENNK.S  R.GFVYRENNK.S | | photosynthesis and photorespiration | | chloroplast | | 5.00% | 20281.19 | | | 7.57 | | 19.16/3.86 | | 1127.235  1127.235 | | -0.58645  0.29255 | | 2  2 | | 1  1 | | 2.5666  2.6403 | 0.539  0.4976 | | 0.00046 |
