# Supplementary material for: Dufulin Activates HrBP1 to Produce Antiviral Responses in Tobacco
Source: PLoS One. 2012 May 25;7(5):e37944. doi: 10.1371/journal.pone.0037944 (PMC3360678; doi:10.1371/journal.pone.0037944)
Supplement: Table S2 — The ratio of up-regulation to differentially expressed protein in group E comparing with that in group B in 2-DE. (DOCX) [file pone.0037944.s012.docx]

**Table S2 The ratio of up-regulation to differentially expressed protein in group E comparing with that in group B in 2-DE.**

| Spot no. | 5942 | 6071 | 6138 | 6087 | 6095 | 6257 | 6288 | 6265 | 6287 |
| --- | --- | --- | --- | --- | --- | --- | --- | --- | --- |
| Ratio | 1.28723 | 2.31966 | 1.28514 | 1.31604 | 1.6325 | 1.98999 | 1.29081 | 1.49341 | 1.31604 |
| Spot no. | 6246 | 6291 | 6527 | 6563 | 6545 | 6449 | 6556 | 6395 | 6883 |
| Ratio | 1.49111 | 1.24821 | 1.44689 | 1.35317 | 1.27646 | 1.77561 | 1.28703 | 2.05393 | 1.38414 |
