# Supplementary material for: Dufulin Activates HrBP1 to Produce Antiviral Responses in Tobacco
Source: PLoS One. 2012 May 25;7(5):e37944. doi: 10.1371/journal.pone.0037944 (PMC3360678; doi:10.1371/journal.pone.0037944)
Supplement: Table S3 — Differentially expressed proteins identified by 2-DE and MS. (DOCX) [file pone.0037944.s013.docx]

**Table S3**

| **Protein description** | **Species** | **GI Number** | **Function Category** | **Subcellular location** | **Theoretical MM (Da)** | **pI** | **Experimental MM (kDa)/pI** | **Peptide count** | **Protein Score** | **Protein Score C.I %** | **Best Ion Score** | **ratio** | **tendency** |
| --- | --- | --- | --- | --- | --- | --- | --- | --- | --- | --- | --- | --- | --- |
| **Proteins identified by MALDI-TOF/TOF Spectrometry (ABI 4700 Proteomics Analyzer, US)** | | | | | | | | | | | | | |
| ribulose bisphosphate carboxylase/oxygenase 2 (RuBisCO activase) | Nicotiana tabacum | gi\|12643758 | photosynthesis and photorespiration | chloroplast | 48312.5 | 8.14 | 39.14/4.40 | 9 | 217 | 100 | 76 | 1.28723 | down-regulation |
| lignin-forming anionic peroxidase | Nicotiana tabacum | gi\|129837 | disease resistance | cytoplasm | 34652.1 | 4.69 | 36.24/4.25 | 8 | 260 | 100 | 142 | 2.31966 | down-regulation |
| photosystem II stability/assembly factor HCF 136 | Ricinus communis | gi\|255559812 | photosynthesis and photorespiration | chloroplast | 43414.9 | 7.11 | 36.01/5.40 | 9 | 209 | 100 | 99 | 1.28514 | down-regulation |
| ribulose-bisphosphate carboxylase activase | Nicotiana tabacum | gi\|100380 | photosynthesis and photorespiration | chloroplast | 25913 | 5.01 | 36.67/6.25 | 8 | 302 | 100 | 117 | 1.31604 | up-regulation |
| NAD dependent epimerase/dehydratase | Ricinus communis | gi\|255542956 | redox metabolism | cytoplasm | 42547 | 8.52 | 66.35/6.75 | 8 | 364 | 100 | 110 | 1.6325 | up-regulation |
| plastid-lipid-associated protein | Nicotiana tabacum | gi\|2632088 | disease resistance | chloroplast | 29385.4 | 4.83 | 32.00/4.55 | 8 | 377 | 100 | 89 | 1.98999 | up-regulation |
| oxygen evolving complex 33 kDa photosystem II protein | Nicotiana tabacum | gi\|30013657 | photosynthesis and photorespiration | chloroplast | 35176.9 | 5.63 | 30.67/4.90 | 15 | 653 | 100 | 170 | 1.29081 | up-regulation |
| predicted protein | Micromonas pusilla | gi\|226461019 | No annotation | No annotation | 272410.5 | 5.67 | 32.67/5.44 | 32 | 75 | 97.645 | / | 1.49341 | down-regulation |
| protein binding protein | Ricinus communis | gi\|255564826 | No annotation | No annotation | 37785.1 | 7.56 | 31.33/5.48 | 8 | 136 | 100 | 60 | 1.31604 | down-regulation |
| predicted protein | Physcomitrella patens subsp. Patens | gi\|168050023 | No annotation / | No annotation | 70926.8 | 9.06 | 33.34/5.78 | 19 | 78 | 98.972 | / | 1.49111 | down-regulation |
| chain A of ribulose 1,5-Biphosphate carboxylase oxygenase | Nicotiana tabacum | gi\|515239 | photosynthesis and photorespiration | chloroplast | 49526.1 | 6.19 | 31.15/7.84 | 11 | 85 | 99.78 | 27 | 1.24821 | up-regulation |
| harpin binding protein 1 | Nicotiana tabacum | gi\|38679323 | disease resistance | cell membrane | 29980 | 8.8 | 34.4/4.66 | 8 | 197 | 100 | 73 | 1.36946 |  |
| oxygen-evolving enhancer protein 2-1 | Nicotiana tabacum | gi\|52000814 | photosynthesis and photorespiration | chloroplast | 28634.4 | 6.84 | 26.16/4.75 | 7 | 315 | 100 | 109 | 1.44689 | up-regulation |
| superoxide dismutase | Nicotiana plumbaginifolia | gi\|134642 | disease resistance | chloroplast | 23027.5 | 5.53 | 25.40/5.66 | 9 | 323 | 100 | 78 | 1.35317 | down-regulation |
| predicted protein | Populus trichocarpa | gi\|224104053 | No annotation | No annotation | 70270.2 | 7.85 | 25.81/5.74 | 14 | 64 | 72.963 | 6 | 1.27646 | down-regulation |
| triose phosphate isomerase cytosolic isoform-like | Solanum tuberosum | gi\|77745458 | energy metabolism | cytoplasm | 26995.5 | 5.73 | 27.75/6.40 | 9 | 376 | 100 | 102 | 1.77561 | up-regulation |
| photosystem I light-harvesting chlorophyll a/b-binding protein | Nicotiana plumbaginifolia | gi\|493723 | photosynthesis and photorespiration | chloroplast | 26319.5 | 5.83 | 25.46/6.90 | 2 | 58 | 0 | 50 | 1.28703 | up-regulation |
| PSI-D1 precursor | Nicotiana sylvestris | gi\|407769 | photosynthesis and photorespiration | thylakoid membrane | 23441.2 | 9.84 | 23.05/7.46 | 9 | 106 | 99.998 | 45 | 1.50195 | down-regulation |
| harpin binding protein 1 | Nicotiana tabacum | gi\|38679323 | disease resistance | cell membrane | 29980 | 8.8 | 28.55/5.66 | 9 | 201 | 100 | 66 | 2.05393 | down-regulation |
| chain s of ribulose-1,5-bisphosphate carboxylase oxygenase | Nicotiana tabacum | gi\|230922 | photosynthesis and photorespiration | chloroplast | 14550.2 | 5.19 | 19.95/4.15 | 7 | 213 | 100 | 68 | 1.6636 | up-regulation |
| putative ribulose bisphosphate carboxylase small subunit protein precursor | Nicotiana tabacum | gi\|30013663 | photosynthesis and photorespiration | chloroplast | 20268.1 | 7.57 | 18.75/3.55 | 7 | 162 | 100 | 27 | 1.38414 | up-regulation |
