# Supplementary material for: Dufulin Activates HrBP1 to Produce Antiviral Responses in Tobacco
Source: PLoS One. 2012 May 25;7(5):e37944. doi: 10.1371/journal.pone.0037944 (PMC3360678; doi:10.1371/journal.pone.0037944)
Supplement: Table S4 — Sequence data (FASTA) of the differentially expressed proteins obtained from MS. (DOCX) [file pone.0037944.s014.docx]

**Table S4**

| >gi\|19992\|emb\|CAA78704.1\| ribulose bisphosphate carboxylase activase [Nicotiana tabacum] |
| --- |
| KLISQRYREAAEIIRKGNMCCLFINDLDAGAGRMGGTTQYTVNNQMVNATLMNIADNPTNVQLPGMYNKQ |
| ENARVPIIVTGNDFSTLYAPLIRDGRMEKFYWAPTREDRIGVCKGIFRTDNVPEEAVVKIVDTFPGQSID |
| FFGALRARVYDDEVRKWVSGTGIEAIGDKLLNSFDGPPTFEQPKMTVEKLLEYGNMLVQEQENVKRVQLA |
| ETYLKEAALGDANADAINTGNF |
|  |
| >gi\|100380\|pir\|\|S25484 ribulose-bisphosphate carboxylase activase (EC 6.3.4.-) (clone TA1.1) - common tobacco (fragment) |
| KLISQRYREAAEIIRKGNMCCLFINDLDAGAGRMGGTTQYTVNNQMVNATLMNIADNPTNVQLPGMYNKQ |
| ENARVPIIVTGNDFSTLYAPLIRDGRMEKFYWAPTREDRIGVCKGIFRTDNVPEEAVVKIVDTFPGQSID |
| FFGALRARVYDDEVRKWVSGTGIEAIGDKLLNSFDGPPTFEQPKMTVEKLLEYGNMLVQEQENVKRVQLA |
| ETYLKEAALGDANADAINTGNF |
|  |
| >gi\|129837\|sp\|P11965.1\|PERX_TOBAC RecName: Full=Lignin-forming anionic peroxidase; AltName: Full=TOPA; Flags: Precursor |
| MSFLRFVGAILFLVAIFGASNAQLSATFYDTTCPNVTSIVRGVMDQRQRTDARAGAKIIRLHFHDCFVNG |
| CDGSILLDTDGTQTEKDAPANVGAGGFDIVDDIKTALENVCPGVVSCADILALASEIGVVLAKGPSWQVL |
| FGRKDSLTANRSGANSDIPSPFETLAVMIPQFTNKGMDLTDLVALSGAHTFGRARCGTFEQRLFNFNGSG |
| NPDLTVDATFLQTLQGICPQGGNNGNTFTNLDISTPNDFDNDYFTNLQSNQGLLQTDQELFSTSGSATIA |
| IVNRYAGSQTQFFDDFVSSMIKLGNISPLTGTNGQIRTDCKRVN |
|  |
| >gi\|134642\|sp\|P22302.1\|SODF_NICPL RecName: Full=Superoxide dismutase [Fe], chloroplastic |
| KFELQPPPYPMDALEPHMSSRTFEFHWGKHHRAYVDNLNKQIDGTELDGKTLEDIILVTYNKGAPLPAFN |
| NAAQAWNHQFFWESMKPNGGGEPSGELLELINRDFGSYDAFVKEFKAAAATQFGSGWAWLAYKPEEKKLA |
| LVKTPNAENPLVLGYTPLLTIDVWEHAYYLDFQNRRPDYISIFMEKLVSWEAVSSRLKAATA |
|  |
| >gi\|223593\|prf\|\|0902172A carboxylase/oxygenase,RBP |
| MQVWPPINKKKYETLSYLPDLSQEQLLLEPDYLLKDGWVPCLEFETEHGFVYRENNKSPGYYDGRYWTMW |
| KLPMFGCTDATQVLAEVGEAKKAYPEAWIRIIGFDNVRQVQCISFIAYKPEGY |
|  |
| >gi\|230922\|pdb\|3RUB\|S Chain S, Crystal Structure Of The Unactivated Form Of Ribulose-1,5- Bisphosphate Carboxylase(Slash)oxygenase From Tobacco Refined At 2.0-Angstroms Resolution |
| MQVWPPINKKKYETLSYLPDLSQEQLLSEVEYLLKNGWVPCLEFETEHGFVYRENNKSPGYYDGRYWTMW |
| KLPMFGCTDATQVLAEVEEAKKAYPQAWIRIIGFDNVRQVQCISFIAYKPEGY |
|  |
| >gi\|407769\|dbj\|BAA02871.1\| PSI-D1 precursor [Nicotiana sylvestris] |
| MAMASQASLFTPSISTSKTADPRVVAPWKQSASSFSAPKLSKSVVAYRPIKAMAVEKAQSATKEAEPAAP |
| VGFTPPQLDPSTPSPIFGGSTGGLLRKAQVDEFYVITWESPKEQIFEMPTGGAAIMREGPNLLKLARKEQ |
| CLALGTRLRSKYKINYRFYRVFPNGEVQYLHPKDGVYPEKVNPGRQGVGQNFRSIGKNKSPIEVKFTGKQ |
| VYDI |
|  |
| >gi\|493723\|emb\|CAA45523.1\| photosystem I light-harvesting chlorophyll a/b-binding protein [Nicotiana tabacum] |
| MASNTLMSCGIAAVSPSVLSSSYSKFAVAVPVCVGAVSGNSRFSMSAEWMPGQPRPPYLDGSAPGDFGFD |
| PLGLGEVPSNLERYKESELIHCRWAMLAVPGILVPEALGLGNWVKAQEWAAIPGGQATYLGQPVPWGTLP |
| TILAIEFLAIAFVEHQRSMEKDPEKKKYPGGAFDPLGYSKDPKKFEELKVKEIKNGRLALLAFVGFCVQQ |
| SAYPGTGPLENLATHLADPWHNNIGDVIILRGILP |
|  |
| >gi\|515239\|pdb\|1RLD\|A Chain A, Solid-State Phase Transition In The Crystal Structure Of Ribulose 1,5-Biphosphate Carboxylase(Slash)oxygenase |
| LTYYTPEYQTKDTDILAAFRVTPQPGVPPEEAGAAVAAESSTGTWTTVWTDGLTSLDRYKGRCYRIERVV |
| GEKDQYIAYVAYPLDLFEEGSVTNMFTSIVGNVFGFKALRALRLEDLRIPPAYVKTFQGPPHGIQVERDK |
| LNKYGRPLLGCTIKPKLGLSAKNYGRAVYECLRGGLDFTKDDENVNSQPFMRWRDRFLFCAEALYKAEAE |
| TGEIKGHYLNATAGTCEEMIKRAVFARELGVPIVMHDYLTGGFTANTSLAHYCRDNGLLLHIHRAMHAVI |
| DRQKNHGIHFRVLAKALRMSGGDHIHSGTVVGKLEGERDITLGFVDLLRDDFVEQDRSRGIYFTQDWVSL |
| PGVLPVASGGIHVWHMPALTEIFGDDSVLQFGGGTLGHPWGNAPGAVANRVALEACVKARNEGRDLAQEG |
| NEIIREACKWSPELAAACEVWKEIVF |
|  |
| >gi\|2632088\|emb\|CAA75657.1\| Plastid-lipid-Associated Protein [Nicotiana tabacum] |
| FTAQAKNYDKEDEWGPEVEQIKPSGGGVSVAEEEPPKEEPSEIELLKKQLVDSFYGTNRGLSASSETRAE |
| IVELITKLESKNPTPAPTEALPLLNGKWILAYTSFSGLFPLLSRGTLPLVRVEEISQTIDSEAFTVQNSV |
| VFAGPLATTSITTNAKFEVRSPKRVQIKFDEGVIGTPQLTDSIELPENIEFLGQKIDLSPFKGLVNSVQD |
| TASSVAKSISSQPPIKFPISNSNAQSWLLTTYLDHELRISRGDGGSVFVLIKEGSPLLKP |
|  |
| >gi\|7939623\|gb\|AAF70824.1\|AF154423_1 putative beta-galactosidase [Solanum lycopersicum] |
| MERRSGYCLSVIMLVFGVVFLHCLVMTSFAANVTYDHRALVVDGRRRVLISGSIHYPRSTPDMWPDLIQK |
| SKDGGLDVIETYVFWNLHEPVRNQYDFEGRKDLINFVKLVERAGLFVHIRIGPYVCAEWNYGGFPLWLHF |
| IPGIEFRTDNEPFKAEMKRFTAKIVDMIKQENLYASQGGPVILSQIENEYGNGDIESRYGPRAKPYVNWA |
| ASMATSLNTGVPWVMCQQPDAPPSVINTCNGFYCDQFKQNSDKTPKMWTENWTGWFLSFGGPVPYRPVED |
| IAFAVARFFQRGGTFQNYYMYHGGTNFGRTSGGPFIATSYDYDAPLDEYGLINQPKWGHLKDLHKAIKLC |
| EAAMVATEPNVTSLGSNIEVSVYKTDSQCAAFLANTATQSDAAVSFNGNSYHLPPWSVSILPDCKNVAFS |
| TAKINSASTISTFVTRSSEADASGGSLSGWTSVNEPVGISNENAFTRMGLLEQINTTADKSDYLWYSLSV |
| NIKNDEPFLQDGSATVLHVKTLGHVLHAYINGRLSGSGKGNSRHSNFTIEVPVTLVPGENKIDLLSATVG |
| LQNYGAFFDLKGAGITGPVQLKGFKNGSTTDLSSKQWTYQVGLKGEDLGLSNGGSTLWKSQTALPTNQPL |
| IWYKASFDAPAGDTPLSMDFTGMGKGEAWVNGQSIGRFWPAYIAPNDGCTDPCNYRGGYNAEKCLKNCGK |
| PSQLLYHVPRSWLKSSGNVLVLFEEMGGDPTKLSFATREIQSVCSRTSDAHPLPIDMWASEDDARKKSGP |
| TLSLECPHPNQVISSIKFASFGTPQGTCGSFIHGRCSSSNALSIVKKACIGSKSCSLGVSINAFGDPCKG |
| VAKSLAVEASCT |
|  |
| >gi\|12643758\|sp\|Q40565.1\|RCA2_TOBAC RecName: Full=Ribulose bisphosphate carboxylase/oxygenase activase 2, chloroplastic; Short=RuBisCO activase 2; Short=RA 2; Flags: Precursor |
| MATSVSTIGAANKAPLSLNNSVAGTSVPSTAFFGKTLKKVYGKGVSSPKVTNRSLRIAAEEKDADPKKQT |
| YSDRWKGLVQDFSDDQQDIARGKGMVDSLFQAPTGTGTHHAVLQSYEYVSQGLRQYNMDNTLDGFYIAPS |
| FMDKLVVHITKNFLKLPNIKVPLILGVWGGKGQGKSFQCELVFRKMGINPIMMSAGELESGNAGEPAKLI |
| RQRYREAAEIIRKGNICCLFINDLDAGAGRMGGTTQYTVNNQMVNATLMNIADNPTNVQLPGMYNKQENA |
| RVPIIVTGNDFSTLYAPLIRDGRMEKFYWAPTREDRIGVCKGIFRTDNVPEEAVIKIVDTFPGQSIDFFG |
| ALRARVYDDEVRKWVSGTGIEAIGDKLLNSFDGPPTFEQPKMTVEKLLEYGNMLVQEQENVKRVQLAETY |
| LKEAALGDANADAINTGNF |
|  |
| >gi\|14195679\|sp\|P00876.2\|RBL_TOBAC RecName: Full=Ribulose bisphosphate carboxylase large chain; Short=RuBisCO large subunit; Flags: Precursor |
| MSPQTETKASVGFKAGVKEYKLTYYTPEYQTKDTDILAAFRVTPQPGVPPEEAGAAVAAESSTGTWTTVW |
| TDGLTSLDRYKGRCYRIERVVGEKDQYIAYVAYPLDLFEEGSVTNMFTSIVGNVFGFKALRALRLEDLRI |
| PPAYVKTFQGPPHGIQVERDKLNKYGRPLLGCTIKPKLGLSAKNYGRAVYECLRGGLDFTKDDENVNSQP |
| FMRWRDRFLFCAEALYKAQAETGEIKGHYLNATAGTCEEMIKRAVFARELGVPIVMHDYLTGGFTANTSL |
| AHYCRDNGLLLHIHRAMHAVIDRQKNHGIHFRVLAKALRMSGGDHIHSGTVVGKLEGERDITLGFVDLLR |
| DDFVEQDRSRGIYFTQDWVSLPGVLPVASGGIHVWHMPALTEIFGDDSVLQFGGGTLGHPWGNAPGAVAN |
| RVALEACVKARNEGRDLAQEGNEIIREACKWSPELAAACEVWKEIVFNFAAVDVLDK |
|  |
| >gi\|30013657\|gb\|AAP03871.1\| oxygen evolving complex 33 kDa photosystem II protein [Nicotiana tabacum] |
| MAASLQAAATLMQPTKVGVAPARNNLQLRSAQSVSKAFGVEPAAARLTCSLQTELKDLAQKCTDAAKVAG |
| FALATSALVVSGANAEGVPKRLTFDEIQSKTYMEVKGTGTANQCPTIEGGVASFAFKPGKYNAKKFCLEP |
| TSFTVKAESVNKNAPPDFQKTKLMTRLTYTLDEIEGPFEVSSDGTVKFEEKDGIDYAAVTVQLPGGERVP |
| FLFTIKQLVASGKPESFSGEFLVPSYRGSSFLDPKGRGGSTGYDNAVALPAGGRGDEEELEKENVKNTAS |
| STGKITLSVTQCKPETGEVIGVFESIQPSDTDLGAKVPKDVKIQGIWYAQLE |
|  |
| >gi\|30013663\|gb\|AAP03874.1\| putative ribulose bisphosphate carboxylase small subunit protein precursor [Nicotiana tabacum] |
| MASSVLSSAAVATRTNVAQANMVAPFTGLKSAASFPVSRKQNLDITSIASNGGRVQCMQVWPPYGKKKYE |
| TLSYLPDLSEEQLLSEIEYLLKNGWVPCLEFETERGFVYRENNKSPGYYDGRYWTMWKLPMFGCTDATQV |
| LAEVGEAKKAYPEAWIRIIGFDNVRQVQCISFIAYKPEGY |
|  |
| >gi\|31711507\|dbj\|BAC77634.1\| 24K germin like protein [Nicotiana tabacum] |
| MFFQAFFIFSLLFLSSDAAVLDFCVGDLSVPDGPGGYACKKPSAVTANDFVFSGLATPVKLNPLIKAAVT |
| PAFAPQFPGLNGLGISMARLDLAIGGVIPMHTHPGASEVLYVVTGEICAGFISSSDNKVFFKNLKQGDIM |
| VFPQGLLHFQINSGKTTGLAIVSFSSPTPGLQITDFALFANDLATELVQATTFLDAATIKKLKGVLGGTN |
|  |
| >gi\|38679323\|gb\|AAR26483.1\| harpin binding protein 1 [Nicotiana tabacum] |
| MASLLQYSTLPLSNNHCSSSLPSLTCHLSKRSNRNTQKLLEKKKYHIKKSLICQSGIDELAFIELPGTKE |
| AKAELIGSLKLKLLSAVSGLNRGLAASEEDLKKADAAAKELESCAGAVDLSADLDKLQGRWKLIYSSAFS |
| GRTLGGSRPGPPTGRLLPITLGQVFQRIDVLSKDFDNIVELELGAPWPLPPAELTATLAHKFELIGSSTI |
| KITFEKTTVKTTGILSQLPPFEVPRIPDQLRPPSNTGSGEFEVTYIDSDTRVTRGDRGELRVFVIS |
|  |
| >gi\|45544515\|dbj\|BAD12595.1\| truncated N protein [Nicotiana tabacum] |
| RWSYDVFLSFRGEDTRKTFTSHLYEVLNDKGIKTFQDDKRLEYGATIPGELCKAIEESQFAIVVFSENYA |
| TSRWCLNELVKIMECKTRFKQTVIPIFYDVDPSHVRNQKESFAKAFEEHETKYKDDVEGIQRWRIALNEA |
| ANLKGSCDNRDKTDADCIRQIVDQISSKLCKISLSYLQNIVGIDTHLEKIESLLEIGINGVRIMGIWGMG |
| GVGKTTIARAIFDTLLGRMDSSYQFDGACFLKDIKENKRGMHSLQNALLSELLREKANYNNEEDGKHQMA |
| SRLRSKKVLIVLDDIDNKDHYLEYLAGDLDWFGNGSRIIITTRDKHLIEKNDIIYEVTALPDHESIQLFK |
| QHAFGKEVPNENFEKLSLEVVNYAKGLPLALKVWGSLLHNLRLTEWKSAIEHMKNNSYSGIIDKLKISYD |
| GLEPKQQEMFLDIACFLRGEEKDYILQILESCHIGAEYGLRILIDKSLVFISEYNQVQMHDLIQDMGKYI |
| VNFQKDPGERSRLWLAKEVEEVMSNNTGTMAMEAIWVSSYSSTLRFSNQAVKNMKRLRVFNMGRSSTHYA |
| IDYLPNNLRCFVCTNYPWESFPSTFELKMLVHLQLRHNSLRHLWTETKKKNNIAEKEGDGILIEFWGDLQ |
| WAFAVSTE |
|  |
| >gi\|51964984\|ref\|XP_507276.1\| PREDICTED P0605H02.36 gene product [Oryza sativa (japonica cultivar-group)] |
| MARGLAVASLLLVALAVVARPPLALAVKDYPADASAVAKKSPASKADTPTTGKESVAGKTDVVTVAKKSP |
| AGKADTSATYKEYAAAKADAVTVTKKSPAAKADTPTTSKESAAGKANAATVAKKSPAGKADTSATATGKE |
| YAAAKADAITVTKKSPAAKADMPATGKESIAKVDAATVAKESTAGKTGKKAAAKEFTMSGKTNTEADAAT |
| VAKKSLAGKAGTPATGKEYAVTKADAATVAKKSPADKTGKESVVAKADTATVTKKSTAGKTGKKVAAKES |
| PASHKTSMEAATKKSTANKTGTETAAKESTVSGKTDTETAAKESTAPGKTDTTAAVKESTAGKGDAPAMA |
| EKSAAGKAEASAAAKESPTNKADAAAAGPTSGGYQYVNFVIKNPVKAKEKSSDRADGLPIDPTPDGQMMH |
|  |
| >gi\|52000814\|sp\|Q7DM39.2\|PSBP1_TOBAC RecName: Full=Oxygen-evolving enhancer protein 2-1, chloroplastic; Short=OEE2; AltName: Full=23 kDa subunit of oxygen evolving system of photosystem II; AltName: Full=OEC 23 kDa subunit; AltName: Full=23 kDa thylakoid membrane protein; Flags: Precursor |
| MASTQCFLHQHALSSSAARTTSSVSSQRYVSSLKPNQLVCRAQKQSSPQEDDGNSVVVSRRLALTVLIGA |
| AAIGSKVSPADAAYGEAANVFGKPKENTDFLAYNGDGFKLQVPAKWNPSKEVEFPGQVLRYEDNFDSTSN |
| LIVTVTPTDKKSITDYGSPEEFLTQVDFLLGKQAYFGKTDSEGGFESGAVATANLLETSSSTVGGKEYYI |
| LSVLTRTADGDEGGKHQLISATVNGGKLYICKAQAGDKRWFKGARKFVENAATSFSVA |
|  |
| >gi\|76556492\|emb\|CAJ32461.1\| putative chloroplast cysteine synthase 1 precursor [Nicotiana tabacum] |
| DEEEIEVHFICPFLLNYVDVSLAYCCRGPIKLCCSQNQPLKVEGLNIAEDVTQLIGNTPMVYLNTIVKGC |
| VANIAAKLEIMEPCCSVKDRIGFSMISDAEEKGLISPGKTVLVEPTSGNTGIGLAFIAASRGYKLILTMP |
| ASMSLERRVLLKAFGAELVLTDPAKGMKGAVSKAEEILNNTPDAYILQQFDNPANPKIHYETTGPEMWED |
| TKGKIDILVAGIGTGGTISGAGRFLKEQNPNIKIIGVEPTESNVLSGGKPGPHKIQGIGAGFIPGNLDQD |
| VMDEVIEISSDEAVETAKQLALQEGLLVGISSGAAALAAIQVGKRPENAGKLIAVVFPSFGERYLSTILF |
| QSIREECEKMQPES |
|  |
| >gi\|77745458\|gb\|ABB02628.1\| triose phosphate isomerase cytosolic isoform-like [Solanum tuberosum] |
| MGRTFFVGGNWKCNGTSEEIKKIVATLNAGQVPSQDVVEVVVSPPFVFLPLVKNELRSDFHVAAQNCWVK |
| KGGAFTGEVSADMLVNLGIPWVILGHSERRAILGESNEFVGDKVAYALSQGLKVIACVGETLEQRESGST |
| MDVVAAQTKAIAERVKDWSNVVVAYEPVWAIGTGKVASPAQAQEVHAELRKWLQANVSAEVAASTRIIYG |
| GSVSGANCKELAGQPDVDGFLVGGASLKPEFIDIIKAAEVKKSA |
|  |
| >gi\|78102516\|ref\|YP_358657.1\| ATP synthase CF1 alpha subunit [Nicotiana sylvestris] |
| MVTIRADEISNIIRERIEQYNREVKIVNTGTVLQVGDGIARIHGLDEVMAGELVEFEEGTIGIALNLESN |
| NVGVVLMGDGLLIQEGSSVKATGRIAQIPVSEAYLGRVINALAKPIDGRGEISASEFRLIESAAPGIISR |
| RSVYEPLQTGLIAIDSMIPIGRGQRELIIGDRQTGKTAVATDTILNQQGQNVICVYVAIGQKASSVAQVV |
| TTLQERGAMEYTIVVAETADSPATLQYLAPYTGAALAEYFMYRERHTLIIYDDPSKQAQAYRQMSLLLRR |
| PPGREAYPGDVFYLHSRLLERAAKLSSSLGEGSMTALPIVETQSGDVSAYIPTNVISITDGQIFLSADLF |
| NSGIRPAINVGISVSRVGSAAQIKAMKQVAGKLKLELAQFAELEAFAQFASDLDKATQNQLARGQRLREL |
| LKQSQSAPLTVEEQIMTIYTGTNGYLDSLEVGQVRKFLVELRTYLKTNKPQFQEIISSTKTFTEEAEALL |
| KEAIQEQMDRFILQEQA |
|  |
| >gi\|82775180\|emb\|CAI28415.1\| putative cell cycle protein [Nicotiana tabacum] |
| QQQLADIPEDAFGSDETKSEKITESEKGHSYVSQSAIALRYRVMPPPCIRNPYLRDASEIDVDPFGNRRS |
| KCAGFNPVIFGNDGLSRYRSDFHEIEQIGTGNFSRVFKVLKRIDGCMYAVKHSTKQLHQDTDRRKALMEV |
| QALAALGPHENVVGYYSSWFENEHLYIQMELCDHSLSN |
|  |
| >gi\|83281193\|dbj\|BAD15110.2\| similar to CDK5 regulatory subunit-associated protein [Nicotiana tabacum] |
| MASSLSSLSTMLSQPHCAVRIKFPKQYSVRFLSSKLLEVQSTSSRRTSVSLGRSSTFSIKISRNFSQCHS |
| RTSLTSKNQIPTLRDFIPKATQTVSSSDVQQESVMISDVMPRGRIYHETYGCQMNVNDMEIVLSIMKNAG |
| YTESVEVPENAEIIFINTCAIRDNAELKVWQRLNYFWFLKRQWKSNVASGRSQSAHPPKVSVLGCMAERL |
| KDKILDSDKMVDVVCGPDAYRDLPRLLEEVDYGQKGINTLLSLEETYADINPVRISKNSISAFVSVMRGC |
| NNMCSFCIVPFTRGRERSRPVESIVKEVAELWKEGVKEVTLLGQNVNSYNDTSGVENPAEPAVSWELSDG |
| FSSMCKVKHVGLRFADLLDRLATEFPEMRFRYTSPHPKDFPDDLLYVMRDRYNICKSIHLPAQSGSSAVL |
| ERMRRGYTREAYLDLVKKIRDIIPDMGISSDFICGFCGETEEDHEDTLSLVKAVCYDMAYMFAYSMREKT |
| HAHRKYVDDVPDDVKQRRLTELIEAFRGSTGQNYDSKIGTLQLVLVEGPNKRAPDTELIGKSDRGHRVSF |
| TNLPIPDKVDNNGKRNPKIGDYVEVHITKSTRASLFGEALAITKLSSFYSSSHEEAVAFASTD |
|  |
| >gi\|90762161\|gb\|ABD97874.1\| phosphomannomutase [Nicotiana tabacum] |
| MAARKPGLIALFDVDGTLTAPRKEVTPEMLKFMKELRKVVTVGVVGGSDLVKISEQLGKTVTTDYDYCFS |
| ENGLVAHKDGKLIGTQSLKSFLGDEKLKEFINFTLHYIADLDIPIKRGTFIEFRSGMLNVSPIGRDCSQE |
| ERDEFEKYDKVHKIRQTMVSVLREKFAHLNLTFSIGGQISFDVFPQGWDKTYCLRYLEEFNEIHFFGDKT |
| YKGGNDHEIYESERTVGHTVTSPEDTVKQCSEQFLGKDNGSS |
|  |
| >gi\|121309841\|dbj\|BAF44222.1\| malate dehydrogenase like-protein [Iris x hollandica] |
| MAKDPVRVLVTGAAGQIGYALVPMIARGVMLGPDQPVILHMLDIPPAAEALNGVKMELVDAAFPLLKGVI |
| ATTDVVEACTGVSIAVMVGGFPRKEGMERKDVMSKNVSIYKSQASALEKYAAANCKVLVVANPANTNALI |
| LKEYAPSIPEKNITCLTRLDHNRALGQISERLNVQVSDVKNVIIWGNHSSTQYPDVSHAIVKTESGEKPV |
| PELVSDDAWLRGEFITTVQQRGAAIIKARKLSSALSAASSACDHIRDWVLGTPEGTFVSMGVYSDGSYNV |
| PAGLIYSFPVTCRDGQWTIVQGLSVDE |
|  |
| >gi\|168050023\|ref\|XP_001777460.1\| predicted protein [Physcomitrella patens subsp. patens] |
| MGEPGSKSQLDPISRHPIRGLLASMRPELVRRLNVVPIMGLRFRFRLFHPSWYSGNVLLVKRSLKVRRPT |
| RSPAGKHDLWEEFLIGVLLKDKQLQSNDRKEALATKALQEVVNKIDQFDGRNISRYLRCYVREMELNRIS |
| EKKMVELFGLATIPKIRNHITSITDHYGNSWEVFSHALKDEYFLEDVDRVTKKLFLEWIEWPNKNLQATE |
| LLRKFERQYSQLSKLELLLEDKEEDEGLTTKWKNVEDAMGLLTKRERRKDRSNIPKTVQAPTAPVCTTPP |
| TMPTVQPSTSLSKKADMEMEEIIRGMQDLQIKLARLEENTSINNLKNVSKQGYVQRCIWCDDASHTRKDC |
| NEFNNMIRQGVICWKDGKIALKDRDGLLQTNFGKGGMRALVQDYLKDHETAARESTSYGARIDDDLGGST |
| KTSEFWASAVSTIQEGKLPREALLRTAATIRGTTGWEDPVESLSVHAYIAKSQHEALMEEKRQGNFDDTR |
| EGNSCKRQSRGNKAREAASQELPVKDTSASLEEKTRETKDKSKSIAYKLLSDIEAATNLKAVLEERILNT |
| KVEFTLKEVLEITKKKFHDVIIDNIKRKRQLMGETRMSHAIDARIYKDEEEVDIGYK |
|  |
| >gi\|222051768\|dbj\|BAH15357.1\| germin like protein [Nicotiana tabacum] |
| MFKLLFLLAIFILGSNAAVQDFCVADLKGPESPAGYSCKSVANVTVDDFVFSGLSAAGNTASIIKAAVTP |
| AFAAQFPGLNGLGLSAARLDLAPGGVIPFHTHPGASEVLLVVQGSITAGFVSSANAVYLKTLKKGDLMIF |
| PQGLLHFQVSDAGYTSVGFVFSSSSSPGLQITDFALFANDLPTKLVEATTFLDEATIKKLKGVLGGTN |
|  |
| >gi\|226461019\|gb\|EEH58312.1\| predicted protein [Micromonas pusilla CCMP1545] |
| MASTVDAPPYTFVAKTARVSWDSVLDFDATPFTNGTDVSPRDLNALDNLARRLEEAKIEPQTGVEAANAA |
| VNLAKLASILQLSSEYQAMRAEARRTLPDLQAAVAKKDEELDMMDEQMEVMEQRGGRSDPRLEAENEELK |
| IRAEQAERRLAEQRDELAVTQEQLDRAKRDVRDEKSKTEVALQKAKHAEDDVKELVEQIAAERQKNVRAA |
| KDDSLAGSRLQQRNQDVARYQKENKLLADENDRMNMRIEDLMAECVEMSETIVKLDDAAQAWRGREADVE |
| AAADGLRRERDKLAAQLETTKMDLEERTHLLQDLEIKFKEEYERFEREKEDLMIDAKRARHGEGVDGARG |
| FDAGSGRSDLVSPRRRMQITRDPRRPANPDDPRYVEDLEAEVAELRDLRVLLLEAYDQLEHDVGREIDIA |
| LKRQRRQHDALHDKLAAQDEALDIETKRFRSIDKELTHAQEDLAEANERCKKYEAGVYGLSDAMRDLKQT |
| RLRVRAADAQVEDAVALSNQLGRKVEDLIEETRYLRQKAGIPEDATLDLGGFKLKSQVESAQMRALNAQL |
| EREVADLEEDRRRLRNELRYRAKWQGEHAARLGLSARQLGMLEEYADALRYGDDALFNDADDAGSHPGFR |
| ASQGIGARAIRELEDANKTLQQRLAEALDRLQRGETGAGGISMPLIAAGSYGGGDHSQVAELQSQLARAR |
| AEIVRLEDGYNVAMMRSSVQGALVGSGGGGGVASVAASPAAAARAPDSPPRGAATAAAAAAAAAPGAAVD |
| ASAYAEALETIDRLRGERNALHARARSLQSMIDAAPATMGVGASGVVAGVGGADYGASTDVQKMREECVA |
| ADRALAGVVAELQTKERELDELAHETAKYKTGIAELQAVRTALYREHVRARNAWSEERKTLLERAKRAET |
| EAEANRIEAKDATALCERLKPGAESGLKEALSAAHSRLAVLQVREVRLSRAVEAATSAEASFRAEKEELE |
| IDVQEMSRACQERLAFHERRAAEAELRAARCQRELDLCVPRPEHAAVVDANRALQVRFKELLETRVKSAV |
| SSAQLKAAQEDAVTARAQADAASAANAAAQNRVRELSAALDDAADRAGREGLAASAVELRREVAETRAEL |
| EASKRLADLAKRESERLTESKADLESAVLGLEHQARSIQHWSPYDRVGVLAEAKARVHEAQEAERKHLSQ |
| LAVAVSEDKHRAEMEAVAAAEDEAARLRAEVVRAEDRAARAETELGRKNAAHAARDAELLQLRSAVRDME |
| RRSDAAAALARANEDVVRLKGGNAQLKHQVQVAEAEADRLHGDCLRLHRRAQVQENRLYGLREDSRGLMQ |
| AQEAALARLETAVAGRVDLSEAEKWERAILDLRKGAERREELLRRAQETLRGAAERARAAELELASLKNI |
| DRVAREHDGGSPDAAMREIRKLSDELMRAKLAAARAERAAKQANDRAEYLETRERERELETRQMEERALI |
| DKRADADRAEEFSRKVRELQREILTARNVGVGDAKKDGANASATATGAADADDADAVLAAPLPRRRANAE |
| ALAAVGAAAAVGLQPGATSMETQRMVLRQIEAIRALKLRATEAEAKCARATEEARAAAAAVQNAENERDA |
| VQRRLDATIAAGTGADGEHGTGATGDDSAVAQVTAVAQATIARLQELVGEKNAALTRAQAAMSDLRADAL |
| SKQDEDRRTIEKLNDLLFKQDQHKIDAMKEQLEYGGVLGGGGGGGGGGGRTPSKSGAGGGRGKFADKSHE |
| QLLALLSEREQAIEVLTQRFEQQRARHEVSEARLLARAQQKEGEMDRVVAEIDRERARGPSRVLETLVAR |
| LKTQLAQKDKRLAQLKEAIRELEKKLVEAMQKQADGSIARADQRMTEVDQFKAEGADKRASTLAAQLKRA |
| REELATAKEREALWAEERARLAAEARAARETAAKAAREAAVARSAAERTRAQSSSRAAAVAVADRAADRR |
| DDDGVDGEVIERLSRQTADAAANAAAERERAEELEKRVAVLKTQNEKLNKLLRAEREESVAIRSGRPAER |
| SSVSVSAATRGENEPPSGGAKESAEETRRREETLARWEEGVKLRKKAESLAKKLATRTREAEEADKLANK |
| RLDLLNELAKEKTALQSRAAKLAEDVKRGAGGGPDAAAAKELADENEALHREIASLQRVVEVDQAAEIAK |
| LKRRLADTPAPAAAATTTTTGRGVTGDELDATARAAAEQRVRTLEGELLAKDDAALGLRFEAEQSHARAE |
| RLQRRLDRLFRGERERDGGDGASGVAAATRRARELEDVVEALKKVVEKQQSEIAAVRGQAAAKERGIEHA |
| RAAKELRAKVRDLEQEMVGLRQLEKTHKETTQRARRLQAQNEQFRARLQSGADAEMLAAAREDAASTALR |
| LAETRDLLVVAQRDAAAARQDALEAMDAAVRGGGGGGGDAAAEIAALRAENADLKVELDALDPAFFDEVM |
| EMKRAYHEQSRTLDRYEATLADYAERLGVSFTPARRASGER |
|  |
| >gi\|255542956\|ref\|XP_002512541.1\| NAD dependent epimerase/dehydratase, putative [Ricinus communis] |
| MARLITIQQQTQPSFSLLTSSLSSDFNGTRLHTQIQCKRRVWQAKGALQVTASSSKNILIMGGTRFIGVF |
| LSRLLVKEGHQVTLFTRGKAPITQKLPGESDQDYADFSSKVLHLKGDRKDFDFVKSSLSAKGFDVVYDIN |
| GREADEVAPILDALPNLEQFIYCSSAGVYLKSDLLPHSEKDAVDPKSRHKGKLETESLLESSGVNWTSIR |
| PVYIYGPLNYNPVEEWFFHRLKAGRPIPIPNSGIQITQLGHVKDLAKAFIQVLGNEKASKQVFNISGEKY |
| VTFDGLARACAKAGGFPEPEIVHYNPKEFDFGKKKAFPFRDQHFFASVDKAKHVLGWEPEFDLVEGLADS |
| YNLDFGRGTFRKEADFTTDDMILGKSLVLQS |
|  |
| >gi\|255559812\|ref\|XP_002520925.1\| Photosystem II stability/assembly factor HCF136, chloroplast precursor, putative [Ricinus communis] |
| MASSTSAFTANIPFLNKSHSHSQSRFVPKASLNNVHLPHHHSLNRRQFLSQTATVSLPLLSSPLIIQQPA |
| NAEETLSEWERVYLPSDPGVVLLDIAFVPDDMNHGFLLGTRQTILETKDGGNTWVSRSIPSAEDEDFNYR |
| FNSISFKGKEGWIVGKPAILLYTSDAGDSWQRIPLSAQLPGDMVYIKATGEKSAEMVTDQGAIYITSNGG |
| YNWRAAVQETVSATLNRTVSSGISGASYYTGTFNTVNRSPDGNYVAVSSRGNFYLTWEPGQPFWQPHNRA |
| VARRIQNMGWRADGGLWLLVRGGGLYLSKGTGISEDFEEISVQSRGFGILDVGYRSKDEAWAAGGSGILL |
| RTTNGGKTWTRDKAADSIAANLYSVKFINDSKGFVLGNDGVLLRYLG |
|  |
| >gi\|255564826\|ref\|XP_002523407.1\| protein binding protein, putative [Ricinus communis] |
| MSLAPSSYASLYTSPPLPRTITIKQNPLIFSQNNPFFSKNCSFFTTSTSFSSTHLLKPSNLTVKASETES |
| KASKSESGSTEGEGEEQYEEYEVELLQPYGIKFAKGRDGATYIDAIAPGGAADKTGMFTVGDKVIATSAV |
| FGTEIWPAAEYGRTMYTIRQRIGPLLMKLQKRYGKTDYAGEMTEKEIIRAERNSGFISDRVREIQMQNYL |
| RKKEQKAQREKDLRDGLILYKNAKYEEALERFESVLGSKPDPNEASVASYNVACCYSKLNQLKAGLSALK |
| DAMEAGFEDFKRIRTDPDLANLRTSEEFEPLLKRFDESFINENAINAIKSLFGFNKK |
