# Supplementary material for: Dufulin Activates HrBP1 to Produce Antiviral Responses in Tobacco
Source: PLoS One. 2012 May 25;7(5):e37944. doi: 10.1371/journal.pone.0037944 (PMC3360678; doi:10.1371/journal.pone.0037944)
Supplement: Table S5 — Gene Ontology (GO) categorization of differentially expressed protein spots based on their molecular function. (DOCX) [file pone.0037944.s015.docx]

**Table S5**

| Level | GO ID | Term | Type | #Seqs | Graph Score | Sequences |
| --- | --- | --- | --- | --- | --- | --- |
| 4 | GO:0046872 | metal ion binding | molecular_function | 2 | 1.2 | gi\|129837\|sp\|P11965.1\|PERX_TOBAC, gi\|30013657\|gb\|AAP03871.1\| |
| 3 | GO:0004871 | signal transducer activity | molecular_function | 3 | 1.8 | gi\|31711507\|dbj\|BAC77634.1\|, gi\|45544515\|dbj\|BAD12595.1\|, gi\|222051768\|dbj\|BAH15357.1\| |
| 3 | GO:0016787 | hydrolase activity | molecular_function | 1 | 1 | gi\|78102516\|ref\|YP_358657.1\| |
| 4 | GO:0004872 | receptor activity | molecular_function | 3 | 3 | gi\|31711507\|dbj\|BAC77634.1\|, gi\|45544515\|dbj\|BAD12595.1\|, gi\|222051768\|dbj\|BAH15357.1\| |
| 2 | GO:0003824 | catalytic activity | molecular_function | 12 | 12.2 | gi\|129837\|sp\|P11965.1\|PERX_TOBAC, gi\|134642\|sp\|P22302.1\|SODF_NICPL, gi\|223593\|prf\|\|0902172A, gi\|230922\|pdb\|3RUB\|S, gi\|515239\|pdb\|1RLD\|A, gi\|14195679\|sp\|P00876.2\|RBL_TOBAC, gi\|30013663\|gb\|AAP03874.1\|, gi\|76556492\|emb\|CAJ32461.1\|, gi\|77745458\|gb\|ABB02628.1\|, gi\|90762161\|gb\|ABD97874.1\|, gi\|121309841\|dbj\|BAF44222.1\|, gi\|78102516\|ref\|YP_358657.1\| |
| 2 | GO:0045735 | nutrient reservoir activity | molecular_function | 2 | 2 | gi\|31711507\|dbj\|BAC77634.1\|, gi\|222051768\|dbj\|BAH15357.1\| |
| 3 | GO:0043167 | ion binding | molecular_function | 2 | 0.43 | gi\|129837\|sp\|P11965.1\|PERX_TOBAC, gi\|30013657\|gb\|AAP03871.1\| |
| 2 | GO:0005488 | binding | molecular_function | 20 | 16.86 | gi\|129837\|sp\|P11965.1\|PERX_TOBAC, gi\|30013657\|gb\|AAP03871.1\|, gi\|19992\|emb\|CAA78704.1\|, gi\|100380\|pir\|\|S25484, gi\|12643758\|sp\|Q40565.1\|RCA2_TOBAC, gi\|45544515\|dbj\|BAD12595.1\|, gi\|78102516\|ref\|YP_358657.1\|, gi\|134642\|sp\|P22302.1\|SODF_NICPL, gi\|493723\|emb\|CAA45523.1\|, gi\|515239\|pdb\|1RLD\|A, gi\|14195679\|sp\|P00876.2\|RBL_TOBAC, gi\|31711507\|dbj\|BAC77634.1\|, gi\|76556492\|emb\|CAJ32461.1\|, gi\|121309841\|dbj\|BAF44222.1\|, gi\|222051768\|dbj\|BAH15357.1\|, gi\|223593\|prf\|\|0902172A, gi\|230922\|pdb\|3RUB\|S, gi\|407769\|dbj\|BAA02871.1\|, gi\|2632088\|emb\|CAA75657.1\|, gi\|30013663\|gb\|AAP03874.1\| |
| 5 | GO:0005509 | calcium ion binding | molecular_function | 2 | 2 | gi\|129837\|sp\|P11965.1\|PERX_TOBAC, gi\|30013657\|gb\|AAP03871.1\| |
| 1 | GO:0003674 | molecular_function | molecular_function | 22 | 20.48 | gi\|129837\|sp\|P11965.1\|PERX_TOBAC, gi\|30013657\|gb\|AAP03871.1\|, gi\|31711507\|dbj\|BAC77634.1\|, gi\|45544515\|dbj\|BAD12595.1\|, gi\|222051768\|dbj\|BAH15357.1\|, gi\|78102516\|ref\|YP_358657.1\|, gi\|134642\|sp\|P22302.1\|SODF_NICPL, gi\|223593\|prf\|\|0902172A, gi\|230922\|pdb\|3RUB\|S, gi\|515239\|pdb\|1RLD\|A, gi\|14195679\|sp\|P00876.2\|RBL_TOBAC, gi\|30013663\|gb\|AAP03874.1\|, gi\|76556492\|emb\|CAJ32461.1\|, gi\|77745458\|gb\|ABB02628.1\|, gi\|90762161\|gb\|ABD97874.1\|, gi\|121309841\|dbj\|BAF44222.1\|, gi\|19992\|emb\|CAA78704.1\|, gi\|100380\|pir\|\|S25484, gi\|12643758\|sp\|Q40565.1\|RCA2_TOBAC, gi\|493723\|emb\|CAA45523.1\|, gi\|407769\|dbj\|BAA02871.1\|, gi\|2632088\|emb\|CAA75657.1\| |
| 3 | GO:0000166 | nucleotide binding | molecular_function | 5 | 5 | gi\|19992\|emb\|CAA78704.1\|, gi\|100380\|pir\|\|S25484, gi\|12643758\|sp\|Q40565.1\|RCA2_TOBAC, gi\|45544515\|dbj\|BAD12595.1\|, gi\|78102516\|ref\|YP_358657.1\| |
| 2 | GO:0016209 | antioxidant activity | molecular_function | 1 | 1 | gi\|129837\|sp\|P11965.1\|PERX_TOBAC |
| 3 | GO:0005515 | protein binding | molecular_function | 6 | 6 | gi\|223593\|prf\|\|0902172A, gi\|230922\|pdb\|3RUB\|S, gi\|407769\|dbj\|BAA02871.1\|, gi\|2632088\|emb\|CAA75657.1\|, gi\|30013663\|gb\|AAP03874.1\|, gi\|45544515\|dbj\|BAD12595.1\| |
| 2 | GO:0060089 | molecular transducer activity | molecular_function | 3 | 1.08 | gi\|31711507\|dbj\|BAC77634.1\|, gi\|45544515\|dbj\|BAD12595.1\|, gi\|222051768\|dbj\|BAH15357.1\| |
| 2 | GO:0005215 | transporter activity | molecular_function | 1 | 1 | gi\|78102516\|ref\|YP_358657.1\| |
| 3 | GO:0016740 | transferase activity | molecular_function | 1 | 1 | gi\|76556492\|emb\|CAJ32461.1\| |
| 4 | GO:0043169 | cation binding | molecular_function | 2 | 0.72 | gi\|129837\|sp\|P11965.1\|PERX_TOBAC, gi\|30013657\|gb\|AAP03871.1\| |
