# Supplementary material for: Dufulin Activates HrBP1 to Produce Antiviral Responses in Tobacco
Source: PLoS One. 2012 May 25;7(5):e37944. doi: 10.1371/journal.pone.0037944 (PMC3360678; doi:10.1371/journal.pone.0037944)
Supplement: Table S6 — GO categorization of differentially expressed proteins based on their localization in various cellular components. (DOCX) [file pone.0037944.s016.docx]

**Table S6**

| Level | GO ID | Term | Type | #Seqs | Graph Score | Sequences |
| --- | --- | --- | --- | --- | --- | --- |
| 2 | GO:0005623 | cell | cellular_component | 25 | 8.09 | gi\|7939623\|gb\|AAF70824.1\|AF154423_1, gi\|493723\|emb\|CAA45523.1\|, gi\|30013657\|gb\|AAP03871.1\|, gi\|45544515\|dbj\|BAD12595.1\|, gi\|255559812\|ref\|XP_002520925.1\|, gi\|78102516\|ref\|YP_358657.1\|, gi\|407769\|dbj\|BAA02871.1\|, gi\|2632088\|emb\|CAA75657.1\|, gi\|52000814\|sp\|Q7DM39.2\|PSBP1_TOBAC, gi\|31711507\|dbj\|BAC77634.1\|, gi\|222051768\|dbj\|BAH15357.1\|, gi\|19992\|emb\|CAA78704.1\|, gi\|100380\|pir\|\|S25484, gi\|134642\|sp\|P22302.1\|SODF_NICPL, gi\|223593\|prf\|\|0902172A, gi\|230922\|pdb\|3RUB\|S, gi\|515239\|pdb\|1RLD\|A, gi\|12643758\|sp\|Q40565.1\|RCA2_TOBAC, gi\|14195679\|sp\|P00876.2\|RBL_TOBAC, gi\|30013663\|gb\|AAP03874.1\|, gi\|76556492\|emb\|CAJ32461.1\|, gi\|83281193\|dbj\|BAD15110.2\|, gi\|77745458\|gb\|ABB02628.1\|, gi\|90762161\|gb\|ABD97874.1\|, gi\|121309841\|dbj\|BAF44222.1\| |
| 2 | GO:0005576 | extracellular region | cellular_component | 3 | 3 | gi\|129837\|sp\|P11965.1\|PERX_TOBAC, gi\|31711507\|dbj\|BAC77634.1\|, gi\|222051768\|dbj\|BAH15357.1\| |
| 3 | GO:0031982 | vesicle | cellular_component | 1 | 0.36 | gi\|7939623\|gb\|AAF70824.1\|AF154423_1 |
| 9 | GO:0016023 | cytoplasmic membrane-bounded vesicle | cellular_component | 1 | 1 | gi\|7939623\|gb\|AAF70824.1\|AF154423_1 |
| 1 | GO:0005575 | cellular_component | cellular_component | 26 | 10.29 | gi\|7939623\|gb\|AAF70824.1\|AF154423_1, gi\|493723\|emb\|CAA45523.1\|, gi\|30013657\|gb\|AAP03871.1\|, gi\|45544515\|dbj\|BAD12595.1\|, gi\|255559812\|ref\|XP_002520925.1\|, gi\|78102516\|ref\|YP_358657.1\|, gi\|407769\|dbj\|BAA02871.1\|, gi\|2632088\|emb\|CAA75657.1\|, gi\|52000814\|sp\|Q7DM39.2\|PSBP1_TOBAC, gi\|31711507\|dbj\|BAC77634.1\|, gi\|222051768\|dbj\|BAH15357.1\|, gi\|19992\|emb\|CAA78704.1\|, gi\|100380\|pir\|\|S25484, gi\|134642\|sp\|P22302.1\|SODF_NICPL, gi\|223593\|prf\|\|0902172A, gi\|230922\|pdb\|3RUB\|S, gi\|515239\|pdb\|1RLD\|A, gi\|12643758\|sp\|Q40565.1\|RCA2_TOBAC, gi\|14195679\|sp\|P00876.2\|RBL_TOBAC, gi\|30013663\|gb\|AAP03874.1\|, gi\|76556492\|emb\|CAJ32461.1\|, gi\|129837\|sp\|P11965.1\|PERX_TOBAC, gi\|83281193\|dbj\|BAD15110.2\|, gi\|77745458\|gb\|ABB02628.1\|, gi\|90762161\|gb\|ABD97874.1\|, gi\|121309841\|dbj\|BAF44222.1\| |
| 2 | GO:0043226 | organelle | cellular_component | 19 | 5.1 | gi\|7939623\|gb\|AAF70824.1\|AF154423_1, gi\|19992\|emb\|CAA78704.1\|, gi\|100380\|pir\|\|S25484, gi\|134642\|sp\|P22302.1\|SODF_NICPL, gi\|223593\|prf\|\|0902172A, gi\|230922\|pdb\|3RUB\|S, gi\|407769\|dbj\|BAA02871.1\|, gi\|493723\|emb\|CAA45523.1\|, gi\|515239\|pdb\|1RLD\|A, gi\|2632088\|emb\|CAA75657.1\|, gi\|12643758\|sp\|Q40565.1\|RCA2_TOBAC, gi\|14195679\|sp\|P00876.2\|RBL_TOBAC, gi\|30013657\|gb\|AAP03871.1\|, gi\|30013663\|gb\|AAP03874.1\|, gi\|52000814\|sp\|Q7DM39.2\|PSBP1_TOBAC, gi\|76556492\|emb\|CAJ32461.1\|, gi\|78102516\|ref\|YP_358657.1\|, gi\|255559812\|ref\|XP_002520925.1\|, gi\|83281193\|dbj\|BAD15110.2\| |
| 3 | GO:0044464 | cell part | cellular_component | 25 | 5.14 | gi\|7939623\|gb\|AAF70824.1\|AF154423_1, gi\|78102516\|ref\|YP_358657.1\|, gi\|407769\|dbj\|BAA02871.1\|, gi\|493723\|emb\|CAA45523.1\|, gi\|2632088\|emb\|CAA75657.1\|, gi\|30013657\|gb\|AAP03871.1\|, gi\|52000814\|sp\|Q7DM39.2\|PSBP1_TOBAC, gi\|255559812\|ref\|XP_002520925.1\|, gi\|31711507\|dbj\|BAC77634.1\|, gi\|222051768\|dbj\|BAH15357.1\|, gi\|19992\|emb\|CAA78704.1\|, gi\|100380\|pir\|\|S25484, gi\|134642\|sp\|P22302.1\|SODF_NICPL, gi\|223593\|prf\|\|0902172A, gi\|230922\|pdb\|3RUB\|S, gi\|515239\|pdb\|1RLD\|A, gi\|12643758\|sp\|Q40565.1\|RCA2_TOBAC, gi\|14195679\|sp\|P00876.2\|RBL_TOBAC, gi\|30013663\|gb\|AAP03874.1\|, gi\|76556492\|emb\|CAJ32461.1\|, gi\|83281193\|dbj\|BAD15110.2\|, gi\|45544515\|dbj\|BAD12595.1\|, gi\|77745458\|gb\|ABB02628.1\|, gi\|90762161\|gb\|ABD97874.1\|, gi\|121309841\|dbj\|BAF44222.1\| |
| 5 | GO:0044424 | intracellular part | cellular_component | 23 | 10.62 | gi\|7939623\|gb\|AAF70824.1\|AF154423_1, gi\|407769\|dbj\|BAA02871.1\|, gi\|493723\|emb\|CAA45523.1\|, gi\|2632088\|emb\|CAA75657.1\|, gi\|30013657\|gb\|AAP03871.1\|, gi\|52000814\|sp\|Q7DM39.2\|PSBP1_TOBAC, gi\|78102516\|ref\|YP_358657.1\|, gi\|255559812\|ref\|XP_002520925.1\|, gi\|19992\|emb\|CAA78704.1\|, gi\|100380\|pir\|\|S25484, gi\|134642\|sp\|P22302.1\|SODF_NICPL, gi\|223593\|prf\|\|0902172A, gi\|230922\|pdb\|3RUB\|S, gi\|515239\|pdb\|1RLD\|A, gi\|12643758\|sp\|Q40565.1\|RCA2_TOBAC, gi\|14195679\|sp\|P00876.2\|RBL_TOBAC, gi\|30013663\|gb\|AAP03874.1\|, gi\|76556492\|emb\|CAJ32461.1\|, gi\|83281193\|dbj\|BAD15110.2\|, gi\|45544515\|dbj\|BAD12595.1\|, gi\|77745458\|gb\|ABB02628.1\|, gi\|90762161\|gb\|ABD97874.1\|, gi\|121309841\|dbj\|BAF44222.1\| |
| 2 | GO:0032991 | macromolecular complex | cellular_component | 5 | 3 | gi\|407769\|dbj\|BAA02871.1\|, gi\|493723\|emb\|CAA45523.1\|, gi\|30013657\|gb\|AAP03871.1\|, gi\|52000814\|sp\|Q7DM39.2\|PSBP1_TOBAC, gi\|78102516\|ref\|YP_358657.1\| |
| 4 | GO:0030312 | external encapsulating structure | cellular_component | 2 | 1.2 | gi\|31711507\|dbj\|BAC77634.1\|, gi\|222051768\|dbj\|BAH15357.1\| |
| 6 | GO:0005737 | cytoplasm | cellular_component | 23 | 10.7 | gi\|7939623\|gb\|AAF70824.1\|AF154423_1, gi\|19992\|emb\|CAA78704.1\|, gi\|100380\|pir\|\|S25484, gi\|134642\|sp\|P22302.1\|SODF_NICPL, gi\|223593\|prf\|\|0902172A, gi\|230922\|pdb\|3RUB\|S, gi\|407769\|dbj\|BAA02871.1\|, gi\|493723\|emb\|CAA45523.1\|, gi\|515239\|pdb\|1RLD\|A, gi\|2632088\|emb\|CAA75657.1\|, gi\|12643758\|sp\|Q40565.1\|RCA2_TOBAC, gi\|14195679\|sp\|P00876.2\|RBL_TOBAC, gi\|30013657\|gb\|AAP03871.1\|, gi\|30013663\|gb\|AAP03874.1\|, gi\|52000814\|sp\|Q7DM39.2\|PSBP1_TOBAC, gi\|76556492\|emb\|CAJ32461.1\|, gi\|78102516\|ref\|YP_358657.1\|, gi\|255559812\|ref\|XP_002520925.1\|, gi\|83281193\|dbj\|BAD15110.2\|, gi\|45544515\|dbj\|BAD12595.1\|, gi\|77745458\|gb\|ABB02628.1\|, gi\|90762161\|gb\|ABD97874.1\|, gi\|121309841\|dbj\|BAF44222.1\| |
| 3 | GO:0043234 | protein complex | cellular_component | 5 | 5 | gi\|407769\|dbj\|BAA02871.1\|, gi\|493723\|emb\|CAA45523.1\|, gi\|30013657\|gb\|AAP03871.1\|, gi\|52000814\|sp\|Q7DM39.2\|PSBP1_TOBAC, gi\|78102516\|ref\|YP_358657.1\| |
| 7 | GO:0044444 | cytoplasmic part | cellular_component | 19 | 11.16 | gi\|7939623\|gb\|AAF70824.1\|AF154423_1, gi\|19992\|emb\|CAA78704.1\|, gi\|100380\|pir\|\|S25484, gi\|134642\|sp\|P22302.1\|SODF_NICPL, gi\|223593\|prf\|\|0902172A, gi\|230922\|pdb\|3RUB\|S, gi\|407769\|dbj\|BAA02871.1\|, gi\|493723\|emb\|CAA45523.1\|, gi\|515239\|pdb\|1RLD\|A, gi\|2632088\|emb\|CAA75657.1\|, gi\|12643758\|sp\|Q40565.1\|RCA2_TOBAC, gi\|14195679\|sp\|P00876.2\|RBL_TOBAC, gi\|30013657\|gb\|AAP03871.1\|, gi\|30013663\|gb\|AAP03874.1\|, gi\|52000814\|sp\|Q7DM39.2\|PSBP1_TOBAC, gi\|76556492\|emb\|CAJ32461.1\|, gi\|78102516\|ref\|YP_358657.1\|, gi\|255559812\|ref\|XP_002520925.1\|, gi\|83281193\|dbj\|BAD15110.2\| |
| 5 | GO:0005618 | cell wall | cellular_component | 2 | 2 | gi\|31711507\|dbj\|BAC77634.1\|, gi\|222051768\|dbj\|BAH15357.1\| |
| 8 | GO:0031410 | cytoplasmic vesicle | cellular_component | 1 | 0.6 | gi\|7939623\|gb\|AAF70824.1\|AF154423_1 |
| 6 | GO:0009579 | thylakoid | cellular_component | 7 | 7 | gi\|407769\|dbj\|BAA02871.1\|, gi\|493723\|emb\|CAA45523.1\|, gi\|2632088\|emb\|CAA75657.1\|, gi\|30013657\|gb\|AAP03871.1\|, gi\|52000814\|sp\|Q7DM39.2\|PSBP1_TOBAC, gi\|78102516\|ref\|YP_358657.1\|, gi\|255559812\|ref\|XP_002520925.1\| |
| 4 | GO:0031988 | membrane-bounded vesicle | cellular_component | 1 | 0.6 | gi\|7939623\|gb\|AAF70824.1\|AF154423_1 |
| 4 | GO:0005622 | intracellular | cellular_component | 23 | 7.37 | gi\|7939623\|gb\|AAF70824.1\|AF154423_1, gi\|78102516\|ref\|YP_358657.1\|, gi\|407769\|dbj\|BAA02871.1\|, gi\|493723\|emb\|CAA45523.1\|, gi\|2632088\|emb\|CAA75657.1\|, gi\|30013657\|gb\|AAP03871.1\|, gi\|52000814\|sp\|Q7DM39.2\|PSBP1_TOBAC, gi\|255559812\|ref\|XP_002520925.1\|, gi\|19992\|emb\|CAA78704.1\|, gi\|100380\|pir\|\|S25484, gi\|134642\|sp\|P22302.1\|SODF_NICPL, gi\|223593\|prf\|\|0902172A, gi\|230922\|pdb\|3RUB\|S, gi\|515239\|pdb\|1RLD\|A, gi\|12643758\|sp\|Q40565.1\|RCA2_TOBAC, gi\|14195679\|sp\|P00876.2\|RBL_TOBAC, gi\|30013663\|gb\|AAP03874.1\|, gi\|76556492\|emb\|CAJ32461.1\|, gi\|83281193\|dbj\|BAD15110.2\|, gi\|45544515\|dbj\|BAD12595.1\|, gi\|77745458\|gb\|ABB02628.1\|, gi\|90762161\|gb\|ABD97874.1\|, gi\|121309841\|dbj\|BAF44222.1\| |
| 8 | GO:0005739 | mitochondrion | cellular_component | 1 | 1 | gi\|83281193\|dbj\|BAD15110.2\| |
| 6 | GO:0043229 | intracellular organelle | cellular_component | 19 | 6.84 | gi\|7939623\|gb\|AAF70824.1\|AF154423_1, gi\|19992\|emb\|CAA78704.1\|, gi\|100380\|pir\|\|S25484, gi\|134642\|sp\|P22302.1\|SODF_NICPL, gi\|223593\|prf\|\|0902172A, gi\|230922\|pdb\|3RUB\|S, gi\|407769\|dbj\|BAA02871.1\|, gi\|493723\|emb\|CAA45523.1\|, gi\|515239\|pdb\|1RLD\|A, gi\|2632088\|emb\|CAA75657.1\|, gi\|12643758\|sp\|Q40565.1\|RCA2_TOBAC, gi\|14195679\|sp\|P00876.2\|RBL_TOBAC, gi\|30013657\|gb\|AAP03871.1\|, gi\|30013663\|gb\|AAP03874.1\|, gi\|52000814\|sp\|Q7DM39.2\|PSBP1_TOBAC, gi\|76556492\|emb\|CAJ32461.1\|, gi\|78102516\|ref\|YP_358657.1\|, gi\|255559812\|ref\|XP_002520925.1\|, gi\|83281193\|dbj\|BAD15110.2\| |
| 8 | GO:0009536 | plastid | cellular_component | 17 | 17 | gi\|19992\|emb\|CAA78704.1\|, gi\|100380\|pir\|\|S25484, gi\|134642\|sp\|P22302.1\|SODF_NICPL, gi\|223593\|prf\|\|0902172A, gi\|230922\|pdb\|3RUB\|S, gi\|407769\|dbj\|BAA02871.1\|, gi\|493723\|emb\|CAA45523.1\|, gi\|515239\|pdb\|1RLD\|A, gi\|2632088\|emb\|CAA75657.1\|, gi\|12643758\|sp\|Q40565.1\|RCA2_TOBAC, gi\|14195679\|sp\|P00876.2\|RBL_TOBAC, gi\|30013657\|gb\|AAP03871.1\|, gi\|30013663\|gb\|AAP03874.1\|, gi\|52000814\|sp\|Q7DM39.2\|PSBP1_TOBAC, gi\|76556492\|emb\|CAJ32461.1\|, gi\|78102516\|ref\|YP_358657.1\|, gi\|255559812\|ref\|XP_002520925.1\| |
| 7 | GO:0043231 | intracellular membrane-bounded organelle | cellular_component | 19 | 11.4 | gi\|7939623\|gb\|AAF70824.1\|AF154423_1, gi\|19992\|emb\|CAA78704.1\|, gi\|100380\|pir\|\|S25484, gi\|134642\|sp\|P22302.1\|SODF_NICPL, gi\|223593\|prf\|\|0902172A, gi\|230922\|pdb\|3RUB\|S, gi\|407769\|dbj\|BAA02871.1\|, gi\|493723\|emb\|CAA45523.1\|, gi\|515239\|pdb\|1RLD\|A, gi\|2632088\|emb\|CAA75657.1\|, gi\|12643758\|sp\|Q40565.1\|RCA2_TOBAC, gi\|14195679\|sp\|P00876.2\|RBL_TOBAC, gi\|30013657\|gb\|AAP03871.1\|, gi\|30013663\|gb\|AAP03874.1\|, gi\|52000814\|sp\|Q7DM39.2\|PSBP1_TOBAC, gi\|76556492\|emb\|CAJ32461.1\|, gi\|78102516\|ref\|YP_358657.1\|, gi\|255559812\|ref\|XP_002520925.1\|, gi\|83281193\|dbj\|BAD15110.2\| |
| 3 | GO:0043227 | membrane-bounded organelle | cellular_component | 19 | 6.84 | gi\|7939623\|gb\|AAF70824.1\|AF154423_1, gi\|19992\|emb\|CAA78704.1\|, gi\|100380\|pir\|\|S25484, gi\|134642\|sp\|P22302.1\|SODF_NICPL, gi\|223593\|prf\|\|0902172A, gi\|230922\|pdb\|3RUB\|S, gi\|407769\|dbj\|BAA02871.1\|, gi\|493723\|emb\|CAA45523.1\|, gi\|515239\|pdb\|1RLD\|A, gi\|2632088\|emb\|CAA75657.1\|, gi\|12643758\|sp\|Q40565.1\|RCA2_TOBAC, gi\|14195679\|sp\|P00876.2\|RBL_TOBAC, gi\|30013657\|gb\|AAP03871.1\|, gi\|30013663\|gb\|AAP03874.1\|, gi\|52000814\|sp\|Q7DM39.2\|PSBP1_TOBAC, gi\|76556492\|emb\|CAJ32461.1\|, gi\|78102516\|ref\|YP_358657.1\|, gi\|255559812\|ref\|XP_002520925.1\|, gi\|83281193\|dbj\|BAD15110.2\| |
