# Supplementary material for: Dufulin Activates HrBP1 to Produce Antiviral Responses in Tobacco
Source: PLoS One. 2012 May 25;7(5):e37944. doi: 10.1371/journal.pone.0037944 (PMC3360678; doi:10.1371/journal.pone.0037944)
Supplement: Table S7 — GO categorization of differentially expressed proteins based on their involvement in various biological processes. (DOCX) [file pone.0037944.s017.docx]

**Table S7**

| Level | GO ID | Term | Type | #Seqs | Graph Score | Sequences |
| --- | --- | --- | --- | --- | --- | --- |
| 2 | GO:0065007 | biological regulation | biological_process | 4 | 1.25 | gi\|31711507\|dbj\|BAC77634.1\|, gi\|45544515\|dbj\|BAD12595.1\|, gi\|222051768\|dbj\|BAH15357.1\|, gi\|30013657\|gb\|AAP03871.1\| |
| 3 | GO:0009056 | catabolic process | biological_process | 3 | 3 | gi\|129837\|sp\|P11965.1\|PERX_TOBAC, gi\|77745458\|gb\|ABB02628.1\|, gi\|121309841\|dbj\|BAF44222.1\| |
| 3 | GO:0051234 | establishment of localization | biological_process | 1 | 0.36 | gi\|78102516\|ref\|YP_358657.1\| |
| 4 | GO:0006629 | lipid metabolic process | biological_process | 1 | 1 | gi\|77745458\|gb\|ABB02628.1\| |
| 4 | GO:0005975 | carbohydrate metabolic process | biological_process | 8 | 8 | gi\|223593\|prf\|\|0902172A, gi\|230922\|pdb\|3RUB\|S, gi\|515239\|pdb\|1RLD\|A, gi\|14195679\|sp\|P00876.2\|RBL_TOBAC, gi\|30013663\|gb\|AAP03874.1\|, gi\|77745458\|gb\|ABB02628.1\|, gi\|90762161\|gb\|ABD97874.1\|, gi\|121309841\|dbj\|BAF44222.1\| |
| 3 | GO:0019748 | secondary metabolic process | biological_process | 1 | 1 | gi\|77745458\|gb\|ABB02628.1\| |
| 4 | GO:0044260 | cellular macromolecule metabolic process | biological_process | 1 | 0.36 | gi\|493723\|emb\|CAA45523.1\| |
| 5 | GO:0019538 | protein metabolic process | biological_process | 1 | 0.36 | gi\|493723\|emb\|CAA45523.1\| |
| 2 | GO:0050896 | response to stimulus | biological_process | 5 | 3 | gi\|31711507\|dbj\|BAC77634.1\|, gi\|222051768\|dbj\|BAH15357.1\|, gi\|129837\|sp\|P11965.1\|PERX_TOBAC, gi\|2632088\|emb\|CAA75657.1\|, gi\|45544515\|dbj\|BAD12595.1\| |
| 7 | GO:0006464 | protein modification process | biological_process | 1 | 1 | gi\|493723\|emb\|CAA45523.1\| |
| 4 | GO:0006139 | nucleobase, nucleoside, nucleotide and nucleic acid metabolic process | biological_process | 2 | 2 | gi\|77745458\|gb\|ABB02628.1\|, gi\|78102516\|ref\|YP_358657.1\| |
| 4 | GO:0006519 | cellular amino acid and derivative metabolic process | biological_process | 1 | 1 | gi\|76556492\|emb\|CAJ32461.1\| |
| 3 | GO:0009719 | response to endogenous stimulus | biological_process | 2 | 2 | gi\|31711507\|dbj\|BAC77634.1\|, gi\|222051768\|dbj\|BAH15357.1\| |
| 5 | GO:0043412 | macromolecule modification | biological_process | 1 | 0.6 | gi\|493723\|emb\|CAA45523.1\| |
| null | GO:0044281 | small molecule metabolic process | biological_process | 1 | 0.6 | gi\|76556492\|emb\|CAJ32461.1\| |
| 3 | GO:0006950 | response to stress | biological_process | 3 | 3 | gi\|129837\|sp\|P11965.1\|PERX_TOBAC, gi\|2632088\|emb\|CAA75657.1\|, gi\|45544515\|dbj\|BAD12595.1\| |
| 3 | GO:0009058 | biosynthetic process | biological_process | 9 | 9 | gi\|223593\|prf\|\|0902172A, gi\|230922\|pdb\|3RUB\|S, gi\|515239\|pdb\|1RLD\|A, gi\|14195679\|sp\|P00876.2\|RBL_TOBAC, gi\|30013663\|gb\|AAP03874.1\|, gi\|76556492\|emb\|CAJ32461.1\|, gi\|77745458\|gb\|ABB02628.1\|, gi\|78102516\|ref\|YP_358657.1\|, gi\|90762161\|gb\|ABD97874.1\| |
| 3 | GO:0044237 | cellular metabolic process | biological_process | 6 | 5.02 | gi\|76556492\|emb\|CAJ32461.1\|, gi\|493723\|emb\|CAA45523.1\|, gi\|30013657\|gb\|AAP03871.1\|, gi\|77745458\|gb\|ABB02628.1\|, gi\|78102516\|ref\|YP_358657.1\|, gi\|121309841\|dbj\|BAF44222.1\| |
| 4 | GO:0006810 | transport | biological_process | 1 | 0.6 | gi\|78102516\|ref\|YP_358657.1\| |
| 4 | GO:0050794 | regulation of cellular process | biological_process | 3 | 1.8 | gi\|31711507\|dbj\|BAC77634.1\|, gi\|45544515\|dbj\|BAD12595.1\|, gi\|222051768\|dbj\|BAH15357.1\| |
| 5 | GO:0006811 | ion transport | biological_process | 1 | 1 | gi\|78102516\|ref\|YP_358657.1\| |
| 6 | GO:0044267 | cellular protein metabolic process | biological_process | 1 | 0.6 | gi\|493723\|emb\|CAA45523.1\| |
| 5 | GO:0007165 | signal transduction | biological_process | 3 | 3 | gi\|31711507\|dbj\|BAC77634.1\|, gi\|45544515\|dbj\|BAD12595.1\|, gi\|222051768\|dbj\|BAH15357.1\| |
| 4 | GO:0008219 | cell death | biological_process | 1 | 1 | gi\|45544515\|dbj\|BAD12595.1\| |
| 3 | GO:0006807 | nitrogen compound metabolic process | biological_process | 2 | 1.2 | gi\|77745458\|gb\|ABB02628.1\|, gi\|78102516\|ref\|YP_358657.1\| |
| 2 | GO:0009987 | cellular process | biological_process | 9 | 4.69 | gi\|76556492\|emb\|CAJ32461.1\|, gi\|493723\|emb\|CAA45523.1\|, gi\|30013657\|gb\|AAP03871.1\|, gi\|77745458\|gb\|ABB02628.1\|, gi\|78102516\|ref\|YP_358657.1\|, gi\|121309841\|dbj\|BAF44222.1\|, gi\|45544515\|dbj\|BAD12595.1\|, gi\|31711507\|dbj\|BAC77634.1\|, gi\|222051768\|dbj\|BAH15357.1\| |
| null | GO:0023060 | signal transmission | biological_process | 3 | 1.8 | gi\|31711507\|dbj\|BAC77634.1\|, gi\|45544515\|dbj\|BAD12595.1\|, gi\|222051768\|dbj\|BAH15357.1\| |
| 2 | GO:0051179 | localization | biological_process | 1 | 0.22 | gi\|78102516\|ref\|YP_358657.1\| |
| 2 | GO:0008152 | metabolic process | biological_process | 16 | 24.14 | gi\|76556492\|emb\|CAJ32461.1\|, gi\|129837\|sp\|P11965.1\|PERX_TOBAC, gi\|134642\|sp\|P22302.1\|SODF_NICPL, gi\|223593\|prf\|\|0902172A, gi\|230922\|pdb\|3RUB\|S, gi\|407769\|dbj\|BAA02871.1\|, gi\|515239\|pdb\|1RLD\|A, gi\|14195679\|sp\|P00876.2\|RBL_TOBAC, gi\|30013663\|gb\|AAP03874.1\|, gi\|52000814\|sp\|Q7DM39.2\|PSBP1_TOBAC, gi\|121309841\|dbj\|BAF44222.1\|, gi\|493723\|emb\|CAA45523.1\|, gi\|30013657\|gb\|AAP03871.1\|, gi\|77745458\|gb\|ABB02628.1\|, gi\|78102516\|ref\|YP_358657.1\|, gi\|90762161\|gb\|ABD97874.1\| |
| 3 | GO:0043170 | macromolecule metabolic process | biological_process | 1 | 0.36 | gi\|493723\|emb\|CAA45523.1\| |
| 3 | GO:0044238 | primary metabolic process | biological_process | 11 | 7.42 | gi\|76556492\|emb\|CAJ32461.1\|, gi\|223593\|prf\|\|0902172A, gi\|230922\|pdb\|3RUB\|S, gi\|515239\|pdb\|1RLD\|A, gi\|14195679\|sp\|P00876.2\|RBL_TOBAC, gi\|30013663\|gb\|AAP03874.1\|, gi\|77745458\|gb\|ABB02628.1\|, gi\|90762161\|gb\|ABD97874.1\|, gi\|121309841\|dbj\|BAF44222.1\|, gi\|493723\|emb\|CAA45523.1\|, gi\|78102516\|ref\|YP_358657.1\| |
| null | GO:0023052 | signaling | biological_process | 3 | 0.65 | gi\|31711507\|dbj\|BAC77634.1\|, gi\|45544515\|dbj\|BAD12595.1\|, gi\|222051768\|dbj\|BAH15357.1\| |
| 3 | GO:0016265 | death | biological_process | 1 | 0.6 | gi\|45544515\|dbj\|BAD12595.1\| |
| 1 | GO:0008150 | biological_process | biological_process | 20 | 18.11 | gi\|76556492\|emb\|CAJ32461.1\|, gi\|31711507\|dbj\|BAC77634.1\|, gi\|222051768\|dbj\|BAH15357.1\|, gi\|129837\|sp\|P11965.1\|PERX_TOBAC, gi\|134642\|sp\|P22302.1\|SODF_NICPL, gi\|223593\|prf\|\|0902172A, gi\|230922\|pdb\|3RUB\|S, gi\|407769\|dbj\|BAA02871.1\|, gi\|515239\|pdb\|1RLD\|A, gi\|14195679\|sp\|P00876.2\|RBL_TOBAC, gi\|30013663\|gb\|AAP03874.1\|, gi\|52000814\|sp\|Q7DM39.2\|PSBP1_TOBAC, gi\|121309841\|dbj\|BAF44222.1\|, gi\|493723\|emb\|CAA45523.1\|, gi\|30013657\|gb\|AAP03871.1\|, gi\|77745458\|gb\|ABB02628.1\|, gi\|78102516\|ref\|YP_358657.1\|, gi\|45544515\|dbj\|BAD12595.1\|, gi\|90762161\|gb\|ABD97874.1\|, gi\|2632088\|emb\|CAA75657.1\| |
| 3 | GO:0050789 | regulation of biological process | biological_process | 4 | 2.08 | gi\|31711507\|dbj\|BAC77634.1\|, gi\|45544515\|dbj\|BAD12595.1\|, gi\|222051768\|dbj\|BAH15357.1\|, gi\|30013657\|gb\|AAP03871.1\| |
| 4 | GO:0006091 | generation of precursor metabolites and energy | biological_process | 5 | 5 | gi\|493723\|emb\|CAA45523.1\|, gi\|30013657\|gb\|AAP03871.1\|, gi\|77745458\|gb\|ABB02628.1\|, gi\|78102516\|ref\|YP_358657.1\|, gi\|121309841\|dbj\|BAF44222.1\| |
| null | GO:0023046 | signaling process | biological_process | 3 | 1.08 | gi\|31711507\|dbj\|BAC77634.1\|, gi\|45544515\|dbj\|BAD12595.1\|, gi\|222051768\|dbj\|BAH15357.1\| |
