# Supplementary material for: Dufulin Activates HrBP1 to Produce Antiviral Responses in Tobacco
Source: PLoS One. 2012 May 25;7(5):e37944. doi: 10.1371/journal.pone.0037944 (PMC3360678; doi:10.1371/journal.pone.0037944)
Supplement: Table S8 — KEGG categorization of differentially expressed proteins based on MS data. (DOCX) [file pone.0037944.s018.docx]

**Table S8**

| Pathway | Seqs in Pathway | Enzyme | Enzyme | NrSeqs | Seqs | PathwayId | PathwayImage |
| --- | --- | --- | --- | --- | --- | --- | --- |
| Phenylpropanoid biosynthesis | 1 | peroxidase | ec:1.11.1.7 | 1 | gi\|129837\|sp\|P11965.1\|PERX_TOBAC | path:map00940 | map00940.gif |
| Glycolysis / Gluconeogenesis | 1 | triose-phosphate isomerase | ec:5.3.1.1 | 1 | gi\|77745458\|gb\|ABB02628.1\| | path:map00010 | map00010.gif |
| Glyoxylate and dicarboxylate metabolism | 6 | malate dehydrogenase | ec:1.1.1.37 | 1 | gi\|121309841\|dbj\|BAF44222.1\| | path:map00630 | map00630.gif |
| Glyoxylate and dicarboxylate metabolism | 6 | ribulose-bisphosphate carboxylase | ec:4.1.1.39 | 5 | gi\|223593\|prf\|\|0902172A, gi\|230922\|pdb\|3RUB\|S, gi\|515239\|pdb\|1RLD\|A, gi\|14195679\|sp\|P00876.2\|RBL_TOBAC, gi\|30013663\|gb\|AAP03874.1\| | path:map00630 | map00630.gif |
| Amino sugar and nucleotide sugar metabolism | 1 | phosphomannomutase | ec:5.4.2.8 | 1 | gi\|90762161\|gb\|ABD97874.1\| | path:map00520 | map00520.gif |
| Cysteine and methionine metabolism | 1 | cysteine synthase | ec:2.5.1.47 | 1 | gi\|76556492\|emb\|CAJ32461.1\| | path:map00270 | map00270.gif |
| Photosynthesis | 1 | H+-transporting two-sector ATPase | ec:3.6.3.14 | 1 | gi\|78102516\|ref\|YP_358657.1\| | path:map00195 | map00195.gif |
| Biosynthesis of plant hormones | 1 | malate dehydrogenase | ec:1.1.1.37 | 1 | gi\|121309841\|dbj\|BAF44222.1\| | path:map01070 | map01070.gif |
| Reductive carboxylate cycle (CO2 fixation) | 1 | malate dehydrogenase | ec:1.1.1.37 | 1 | gi\|121309841\|dbj\|BAF44222.1\| | path:map00720 | map00720.gif |
| Oxidative phosphorylation | 1 | H+-transporting two-sector ATPase | ec:3.6.3.14 | 1 | gi\|78102516\|ref\|YP_358657.1\| | path:map00190 | map00190.gif |
| Sulfur metabolism | 1 | cysteine synthase | ec:2.5.1.47 | 1 | gi\|76556492\|emb\|CAJ32461.1\| | path:map00920 | map00920.gif |
| Citrate cycle (TCA cycle) | 1 | malate dehydrogenase | ec:1.1.1.37 | 1 | gi\|121309841\|dbj\|BAF44222.1\| | path:map00020 | map00020.gif |
| Fructose and mannose metabolism | 2 | triose-phosphate isomerase | ec:5.3.1.1 | 1 | gi\|77745458\|gb\|ABB02628.1\| | path:map00051 | map00051.gif |
| Fructose and mannose metabolism | 2 | phosphomannomutase | ec:5.4.2.8 | 1 | gi\|90762161\|gb\|ABD97874.1\| | path:map00051 | map00051.gif |
| Phenylalanine metabolism | 1 | peroxidase | ec:1.11.1.7 | 1 | gi\|129837\|sp\|P11965.1\|PERX_TOBAC | path:map00360 | map00360.gif |
| Metabolic pathways | 11 | H+-transporting two-sector ATPase | ec:3.6.3.14 | 1 | gi\|78102516\|ref\|YP_358657.1\| | path:map01100 | map01100.gif |
| Metabolic pathways | 11 | triose-phosphate isomerase | ec:5.3.1.1 | 1 | gi\|77745458\|gb\|ABB02628.1\| | path:map01100 | map01100.gif |
| Metabolic pathways | 11 | malate dehydrogenase | ec:1.1.1.37 | 1 | gi\|121309841\|dbj\|BAF44222.1\| | path:map01100 | map01100.gif |
| Metabolic pathways | 11 | phosphomannomutase | ec:5.4.2.8 | 1 | gi\|90762161\|gb\|ABD97874.1\| | path:map01100 | map01100.gif |
| Metabolic pathways | 11 | peroxidase | ec:1.11.1.7 | 1 | gi\|129837\|sp\|P11965.1\|PERX_TOBAC | path:map01100 | map01100.gif |
| Metabolic pathways | 11 | cysteine synthase | ec:2.5.1.47 | 1 | gi\|76556492\|emb\|CAJ32461.1\| | path:map01100 | map01100.gif |
| Metabolic pathways | 11 | ribulose-bisphosphate carboxylase | ec:4.1.1.39 | 5 | gi\|223593\|prf\|\|0902172A, gi\|230922\|pdb\|3RUB\|S, gi\|515239\|pdb\|1RLD\|A, gi\|14195679\|sp\|P00876.2\|RBL_TOBAC, gi\|30013663\|gb\|AAP03874.1\| | path:map01100 | map01100.gif |
| Inositol phosphate metabolism | 1 | triose-phosphate isomerase | ec:5.3.1.1 | 1 | gi\|77745458\|gb\|ABB02628.1\| | path:map00562 | map00562.gif |
| Selenoamino acid metabolism | 1 | cysteine synthase | ec:2.5.1.47 | 1 | gi\|76556492\|emb\|CAJ32461.1\| | path:map00450 | map00450.gif |
| Pyruvate metabolism | 1 | malate dehydrogenase | ec:1.1.1.37 | 1 | gi\|121309841\|dbj\|BAF44222.1\| | path:map00620 | map00620.gif |
| Methane metabolism | 1 | peroxidase | ec:1.11.1.7 | 1 | gi\|129837\|sp\|P11965.1\|PERX_TOBAC | path:map00680 | map00680.gif |
| Biosynthesis of alkaloids derived from terpenoid and polyketide | 1 | malate dehydrogenase | ec:1.1.1.37 | 1 | gi\|121309841\|dbj\|BAF44222.1\| | path:map01066 | map01066.gif |
| Biosynthesis of alkaloids derived from histidine and purine | 1 | malate dehydrogenase | ec:1.1.1.37 | 1 | gi\|121309841\|dbj\|BAF44222.1\| | path:map01065 | map01065.gif |
| Biosynthesis of alkaloids derived from ornithine, lysine and nicotinic acid | 1 | malate dehydrogenase | ec:1.1.1.37 | 1 | gi\|121309841\|dbj\|BAF44222.1\| | path:map01064 | map01064.gif |
| Biosynthesis of alkaloids derived from shikimate pathway | 1 | malate dehydrogenase | ec:1.1.1.37 | 1 | gi\|121309841\|dbj\|BAF44222.1\| | path:map01063 | map01063.gif |
| Biosynthesis of terpenoids and steroids | 1 | malate dehydrogenase | ec:1.1.1.37 | 1 | gi\|121309841\|dbj\|BAF44222.1\| | path:map01062 | map01062.gif |
| Biosynthesis of phenylpropanoids | 2 | malate dehydrogenase | ec:1.1.1.37 | 1 | gi\|121309841\|dbj\|BAF44222.1\| | path:map01061 | map01061.gif |
| Biosynthesis of phenylpropanoids | 2 | peroxidase | ec:1.11.1.7 | 1 | gi\|129837\|sp\|P11965.1\|PERX_TOBAC | path:map01061 | map01061.gif |
| Carbon fixation in photosynthetic organisms | 7 | triose-phosphate isomerase | ec:5.3.1.1 | 1 | gi\|77745458\|gb\|ABB02628.1\| | path:map00710 | map00710.gif |
| Carbon fixation in photosynthetic organisms | 7 | malate dehydrogenase | ec:1.1.1.37 | 1 | gi\|121309841\|dbj\|BAF44222.1\| | path:map00710 | map00710.gif |
| Carbon fixation in photosynthetic organisms | 7 | ribulose-bisphosphate carboxylase | ec:4.1.1.39 | 5 | gi\|223593\|prf\|\|0902172A, gi\|230922\|pdb\|3RUB\|S, gi\|515239\|pdb\|1RLD\|A, gi\|14195679\|sp\|P00876.2\|RBL_TOBAC, gi\|30013663\|gb\|AAP03874.1\| | path:map00710 | map00710.gif |
